# Effects of N6-Methyladenosine Regulators on LAG3 and Immune Infiltrates in Lung Adenocarcinoma

**DOI:** 10.1155/2022/1829528

**Published:** 2022-08-23

**Authors:** Nengchao Wang, Yue Xu, Linzhi Jin, Xiaomin Wang, Shouxin Wu, Yu Wang, Jiangman Zhao, Fuyou Zhou, Hong Ge

**Affiliations:** ^1^Department of Radiation Oncology, The Affiliated Cancer Hospital of Zhengzhou University & Henan Cancer Hospital, Zhengzhou, China; ^2^Department of Radiation Oncology, Anyang Tumor Hospital, The Fourth Affiliated Hospital of Henan University of Science and Technology, Anyang, China; ^3^Shanghai Biotecan Pharmaceuticals Co., Ltd., Shanghai, China; ^4^Shanghai Zhangjiang Institute of Medical Innovation, Shanghai, China

## Abstract

**Background:**

Lung adenocarcinoma (LUAD) is the most common histological subtype of lung cancer, which is one of the most commonly diagnosed tumors and the leading causes of death from cancer around the world. Since RNA methylation is a posttranscriptional modification and affects so much biological progress, it is urged to explore the role of N6-methyladenosine (m6A) methylation in LUAD.

**Methods:**

We explored the expression of 24 m6A methylation genes, as well as their correlations with LAG3 in 561 LUAD samples from TCGA. Consensus clustering was applied to m6A methylation genes, and two LUAD subgroups were identified. The expression of m6A genes was analyzed by the Wilcoxon test. KEGG and GO enrichment analyses were performed to indicate the pathway affected by differentially expressed genes in the two groups. A prognostic model based on LASSO regression using an eleven-m6A gene signature was constructed according to the expression of these genes. Receiver operating characteristic (ROC) curve was used to confirm the accuracy of the model in the TCGA cohort, as well as in the test cohort from the Gene Expression Omnibus (GEO) database.

**Results:**

Compared to cluster 1, cluster 2 showed poorer overall survival (OS) and higher LAG3 expression. In addition, KEGG and GO enrichment analyses indicated that differentially expressed genes are enriched in the immune response. We also observed that the expression of LAG3 is positively correlated with IGF2BP2, CBLL1, and HNRNPA2B1 and negatively correlated with YTHDF2, YTHDF3, and FTO. For patients in the TCGA cohort, the AUC score is 0.7, and the AUC score for the GSE50081 cohort is 0.675. Patients with lower risk scores exhibited better overall survival and lower expression of LAG3 than patients with higher risk scores.

**Conclusions:**

In brief, our results indicated the important role of m6 methylation in affecting the tumor immune microenvironment and the survival of patients with LUAD. The m6A methylation gene signatures might serve as promising therapeutic targets and help the immunotherapy of LUAD in the future.

## 1. Introduction

Lung cancer is one of the most commonly diagnosed tumors and the leading causes of death from cancer. In 2018, approximately 2.09 million cases of lung cancer were newly diagnosed around the world, based on the estimation from the World Health Organization [1]. It is also estimated that lung cancer may lead to about 20% of cancer-related deaths. There are mainly two groups of lung cancer. Nearly 15% of lung cancers are small-cell carcinoma (SCLC), while 85% of lung cancers are non-small-cell carcinoma (NSCLC) [2]. NSCLC can be further classified as adenocarcinoma (AC) and squamous cell carcinoma (SCC), which account for about 40% and 25%-30% of all lung cancers, respectively [3]. Lung adenocarcinoma (LUAD) is usually diagnosed at an advanced stage due to the lack of specific clinical symptoms. Although the treatment of LUAD combines surgical resection, chemotherapy, radiation therapy, and targeted therapy, the five-year relative survival rate of LUAD is still less than 20% [4]. Thus, it is important to accurately predict the prognosis of patients with lung cancer. So far, histopathology is being used, but it has the limitations that the same pathology may indicate a different prognosis because of individual differences. Thus, it is necessary to focus on molecular biomarkers as they might improve the prognosis and treatment of LUAD [5].

Messenger RNA (mRNA) modification is a crucial regulator at the posttranscriptional level, and N6-methyladenosine (m6A) methylation is the most abundant type of mRNA modification [6]. RNA methylation, a biochemical process that introduces a methyl group (-CH3) to an RNA molecular, is regulated by a family of enzymes called methyltransferases, which are also called “writers.” The encoding genes of the components of “writer” include methyltransferase-like 3 (METTL3) [7], METTL14 [8], METTL16 [9], Wilms tumor 1-associated protein (WTAP) [10], RNA-binding motif protein 15/15B (RBM15/15B) [11], zinc finger CCCH domain-containing protein 13 (ZC3H13) [12], and KIAA1429 [13]. Methylation is a reversible process, and the enzymes that remove methyl groups (demethylation) are called demethylases or “erasers.” These enzymes are encoded by fat mass and obesity-associated (FTO) and alkB homolog 5 (ALKBH5) [14, 15]. There is another family of enzymes that neither add nor remove the methyl group but recognize the modifications and perform different biological functions, so as to pass the regulation signal downstream. Therefore, they are called “readers,” including heterogeneous nuclear ribonucleoprotein C (HNRNPC) [16], YTH domain families (YTHDC1, YTHDC2, YTHDF1, YTHDF2, and YTHDF3) [17], IGF2 mRNA binding protein (IGF2BP) families (IGF2BP1, IGF2BP2, and IGF2BP3) [18], fragile X mental retardation 1 (FMR1) [19], leucine-rich pentatricopeptide repeat containing (LRPPRC) [20], Casitas B-lineage lymphoma-transforming sequence-like protein 1 (CBLL-1) [21], ELAV-Like RNA Binding Protein 1 (ELAVL1) [22], and heterogeneous nuclear ribonucleoprotein A2/B1 [23].

Recent evidence has shown the associations between m6A modification and tumor proliferation, differentiation, and genesis. For example, the high expression of FTO may lead to leukemic cell transformation and tumorigenesis in acute myeloid leukemia [24], and it is observed that the expression of FTO and METTL3 were upregulated in renal clear cell carcinoma. In hepatocellular carcinoma (HCC), the upregulation of METTL3 [25] and downregulation of METTL14 [26] may contribute to HCC progression and metastasis, causing a poor prognosis of patients. It is also reported that FTO is associated with the progression of lung cancer by increasing ubiquitin-specific protease (USP7) expression [27], and m6A is involved in afatinib resistance in NSCLC [28]. Except for the changes in the transcript level of certain m6A genes, the perturbation of overall m6A level was also reported in many types of cancers, including colorectal cancer [29], adrenocortical carcinoma [30], gastric cancer, and hepatocellular carcinoma [31].

In addition, more and more studies revealed the close relationship between m6A and immune responses. For example, m6A modifications may reduce the type I interferon production during the antiviral innate immune response [32]. Knockout of METTL3 and METTL14 could inhibit the IL-7-JAK1/STAT5 signaling pathway and therefore increase the production of Th1 and Th17 [33]. A study by Tong et al. reported that severe systemic autoimmune diseases were triggered in conditional METTL3-knockout mice, suggesting the abnormal function of Tregs [34]. The connection between m6A and immune response may partially explain the role of m6A in cancer, as demonstrated in some studies. Three m6A modification patterns were identified in colon cancer, and they are highly consistent with the three known immune infiltration profiles (immune-inflamed, immune-excluded, and immune-desert) [35]. Moreover, the infiltration of M1/M2-like tumor-associated macrophages and regulatory T cells in tumors was observed in METTL3-deficient lung cancer and melanoma mice model [36]. ALKBH5 can directly interact with programmed cell death 1 (PD-L1) mRNA and may promote the expression of PD-L1 and inhibit the expansion and cytotoxicity of T cells [37]. In addition, it is reported that METTL3 mediates the m6A modification of circular RNA circIGF2BP3 and promotes its circularization in a YTHDC1-dependent manner in LUAD, causing the upregulation of PKP3 and PD-L1, leading to the attenuation of immune response [38]. Meanwhile, METTL3-IGF2BP3-dependent PD-L1 mRNA activation was also observed in breast cancer cells [39].

Although a significant relationship was suggested among m6A, cancer, and immune response, a clear understanding of the function of m6A modification in lung cancer, especially its role in immune regulation, has not been achieved. Thus, in this study, we evaluated the 24 m6A modification gene expressions in samples from TCGA and described the immune landscape of the two clusters defined by consensus clustering. Prognostic signatures based on 11 m6A methylation genes were also established to potentially improve the clinical treatment decision. Our study also analyzed and discussed the correlation between lymphocyte-activation gene 3 (LAG3) and m6A methylation genes to seek their associations with the prognosis of LUAD.

## 2. Materials and Methods

### 2.1. Data Acquisition from the TCGA Datasets

The Cancer Genome Atlas (TCGA) LUAD datasets (*n* = 561) were downloaded from the UCSC Xena browser (https://xenabrowser.net/). The gene expression data were presented as FPKM values derived from TCGA level 3 data. 502 tumor and 59 normal samples were acquired after filtering samples without survival time and status.

### 2.2. m6A Methylation Gene Selection

We selected 24 m6A methylation genes that were commonly discussed in several research articles and reviews, namely, METTL3, METTL14, METTL16, RBM15/15B, WTAP, ZC3H13, KIAA1429, HNRNPC, YTHDC1/2, YTHDF1/2/3, IGF2BP1/2/3, FMR1, LRPPRC, CBLL1, ELAVL1, HNRNPA2B1, ALKBH5, and FTO.

### 2.3. Bioinformatic Analysis and Statistical Analysis

The differential expression analysis was conducted by the Wilcoxon rank-sum test while comparing the expression of m6A methylation genes between 2 groups. The Kruskal-Wallis test was applied for the comparison among multiple groups. Differential expression genes were identified with *p* value < 0.05. All statistical analyses were performed using R language (version 3.6.1) (http://mirrors.tuna.tsinghua.edu.cn/CRAN/). To quantify the proportion of m6A gene expression in LUAD samples, we conducted an unsupervised clustering analysis and classified LUAD into 2 clusters using “ConsensusClusterPlus” (100 iterations, 80% resampling rate Pearson correlation, http://www.bioconductor.org/). The edgeR package in R was used to identify differentially expressed genes (DEGs) in both cluster 1/2 and immunity high/low group. The *t*-test method was used to calculate the *p* value. The DEGs were screened out with the threshold of *p* value < 0.05 and |*logFC*| > 1.

### 2.4. Survival Analysis

To evaluate and compare the overall survival of patients with LUAD in different subgroups in our study, the OS probability of each LUAD sample was estimated by the Kaplan-Meier method. The log-rank test was used to determine the significance of OS probability between different categorical LUAD groups. Also, in this study, the clusterProfiler package was used to identify and visualize the GO terms and KEGG pathways enriched by DEGs. *p* value < 0.05 was set as the cut-off criterion for the significant enrichment.

For each LUAD dataset, we quantified the enrichment levels of 29 immune signatures in each sample, by the single-sample gene-set enrichment analysis (ssGSEA) score, as described in He et al.'s research [40]. Based on the enrichment levels (ssGSEA scores) of these 29 immune signatures, we performed hierarchical clustering for patients with LUAD. We also applied a Cox regression analysis on the immune cells in data from TCGA to find cell types that were significantly correlated with OS. CIBERSORT (https://cibersort.stanford.edu/) was used to estimate the fraction of 22 immune cell types by using the corresponding RNA transcripts subsets.

LASSO regression analysis was conducted in the TCGA cohort to establish the prognostic risk signature. We calculated and selected the optimal penalty parameter *λ* that was correlated with the minimum 10-fold cross-validation to screen the signatures. The risk score was calculated by adding up all the coefficients obtained from the LASSO regression algorithm. To be specific, risk score = sum of coefficients of different m6A methylation genes × the expression level of corresponding m6A methylation genes. In order to evaluate the predictive performance of this model, concordance index (C-index) that represents the fraction of patients whose predicted survival times are correctly ordered and the calibration curves that assess the consistency of predicted survival and the actual survival were calculated and performed with the rms package in R. According to the cut-off point, which is the median value of all the risk scores, patients were designated to the high-risk group and low-risk group for subsequent analysis.

## 3. Results

### 3.1. Expression of m6A Genes in LUAD Group, Normal Group, and Different Tumor Stages

**Figure 1:**
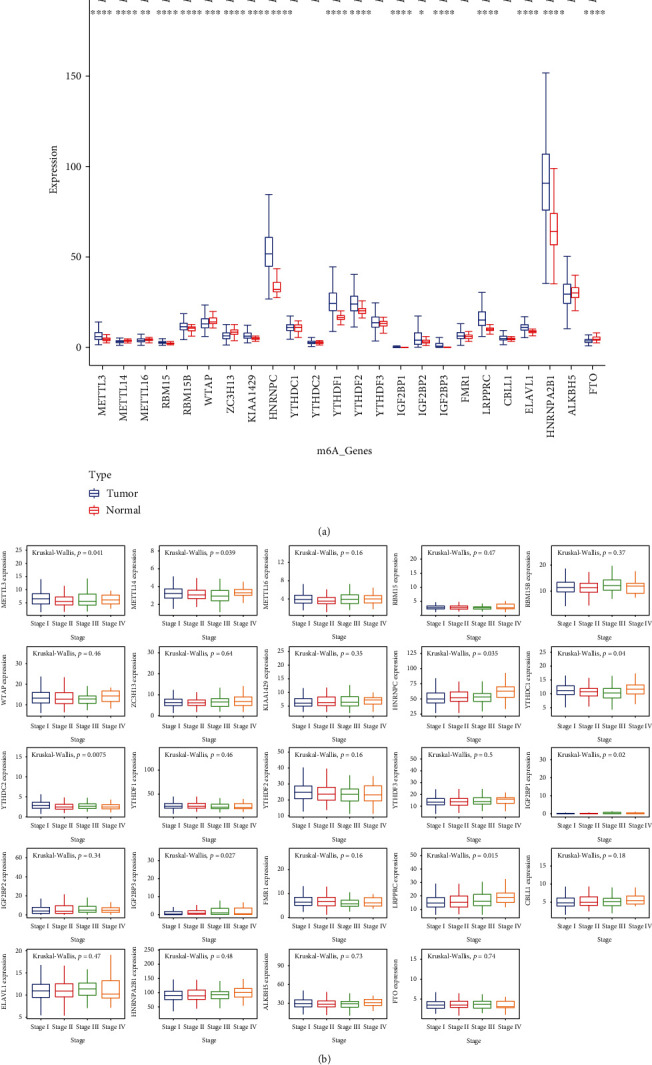
(a) The expression of m6A methylation genes in tumor and healthy samples. (b) The expression of m6A methylation genes in different tumor stages.

Comparing the expression of 24 m6A genes between 502 tumor samples and 59 normal samples, statistical differences were found in 18 of these genes. Four genes were downregulated, namely, METTL14, WTAP, ZC3H13, and FTO, while 14 genes were upregulated, including METTL3, METTL16, RBM15, RBM15B, KIAA1429, HNRNPC, YTHDF1, YTHDF2, IGF2BP1, IGF2BP2, IGF2BP3, LRPPRC, ELAVL1, and HNRNPA2B1 (Figure 1(a)). The expression of these m6A genes in different tumor stages was also analyzed. As is shown in Figure 1(b), there were 8 genes expressed differently in different stages. To be specific, these genes were METTL3, METTL14, HNRNPC, YTHDC1, YTHDC2, IGF2BP1, IGF2BP3, and LRPPRC. It is worth noting that HNRNPC and LRPPRC were continuously upregulated from stage I to IV. All these genes were also tested for the relevance between the expression level and survival. The Kaplan-Meier curves (Supplementary Figure 1) indicate that the low expression of ELAVL1, HNRNPA2B1, HNRNPC, IGF2BP1, IGF2BP2, IGF2BP3, KIAA1429, and RBM15 was related with better overall survival (OS). Meanwhile, the high expression of YTHDC2 and YTHDF2 has positive effects on OS.

### 3.2. Consensus Clustering of m6A Genes Revealed the Difference in Immune-Related Pathways in Two Clusters of LUAD

**Figure 2:**
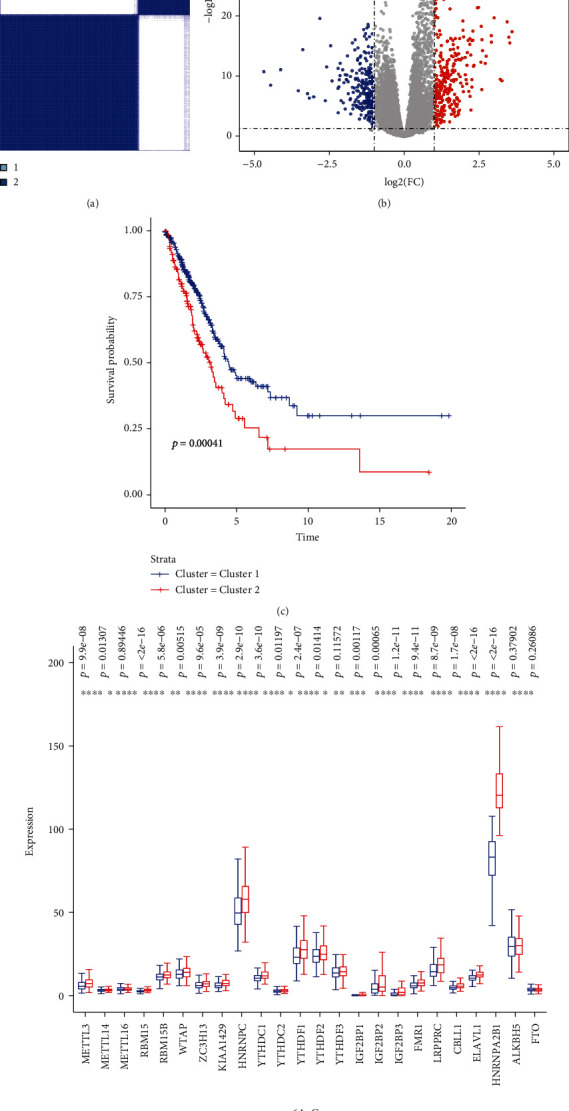
(a) Consensus clustering matrix for *k* = 2. (b) Differentially expressed genes (DEGs) in clusters 1 and 2. With the threshold set on *p* value < 0.05 and |*logFC*| > 1. (c) Kaplan-Meier curves of overall survival for patients in clusters 1 and 2. (d) The expression of m6A methylation genes in cluster 1 and cluster 2.

**Figure 3:**
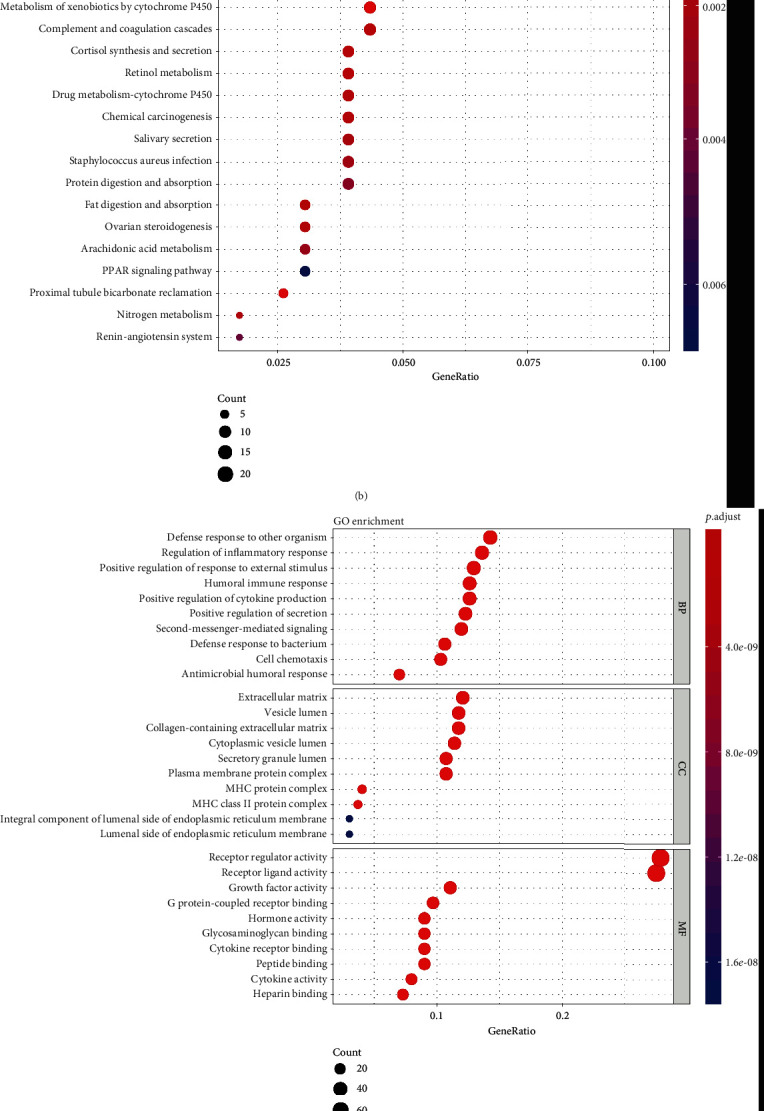
(a and b) GO (a) and KEGG (b) enrichment analyses for DEGs in clusters 1 and 2. (c and d) GO (c) and KEGG (d) enrichment analyses for genes that belonged to DEGs in clusters 1 and 2 and were listed in ImmPort and InnateDB.

To get a better understanding of the biological function of m6A gene expression in LUAD, consensus clustering was performed based on the gene expression of 24 m6A methylation genes. According to the analysis, the optimal *k* is 2, and therefore, two subgroups were clustered, namely, cluster 1 and cluster 2 (Figure 2(a)). The comparison of the m6A gene expression between these 2 clusters is shown in Figure 2(d). Significant differences and clear distinctions were found in most of these genes. It is worth mentioning that cluster 2 showed a unignorably high expression of HNRNPA2B1. To further identify the meaning of this clustering, the Kaplan-Meier curves representing the OS situation of these two groups were generated, and a significant difference (*p* = 0.00041) was observed (Figure 2(c)). Then, an R package named “edgeR” was used to identify DEGs in genome-wide between these 2 clusters. With the threshold set on *p* value < 0.05 and |*logFC*| > 1, a total of 608 DGEs were found (Figure 2(b)). Their biological functions might be the major reason that cluster 1 had a better OS than cluster 2, so Kyoto Encyclopedia of Genes and Genomes (KEGG) and Gene Ontology (GO) enrichment analyses were also performed. Interestingly, many biological processes enriched by GO analysis were related to immune response, especially humoral response (Figure 3(a)). KEGG pathway enrichment analysis indicated that DEGs were mainly enriched in neuroactive ligand-receptor interaction and several metabolism pathways (Figure 3(b)). To get a clearer view of how different expression patterns of m6A methylation genes may affect immune-related pathways, we perform the KEGG and GO enrichment analyses again using the DEGs that are listed in ImmPort and InnateDB. The selected DEGs are shown in Supplementary Figure 2 and Supplementary Table 1. In addition to humoral immune response and defense against other organisms, regulation of inflammatory response and positive regulation of cytokine production were also enriched by GO analysis (Figure 3(c)). The second KEGG analysis mainly enriched the cytokine-cytokine receptor interaction pathway (Figure 3(d)).

### 3.3. Different Immune Landscapes in the Patients with LUAD

**Figure 4:**
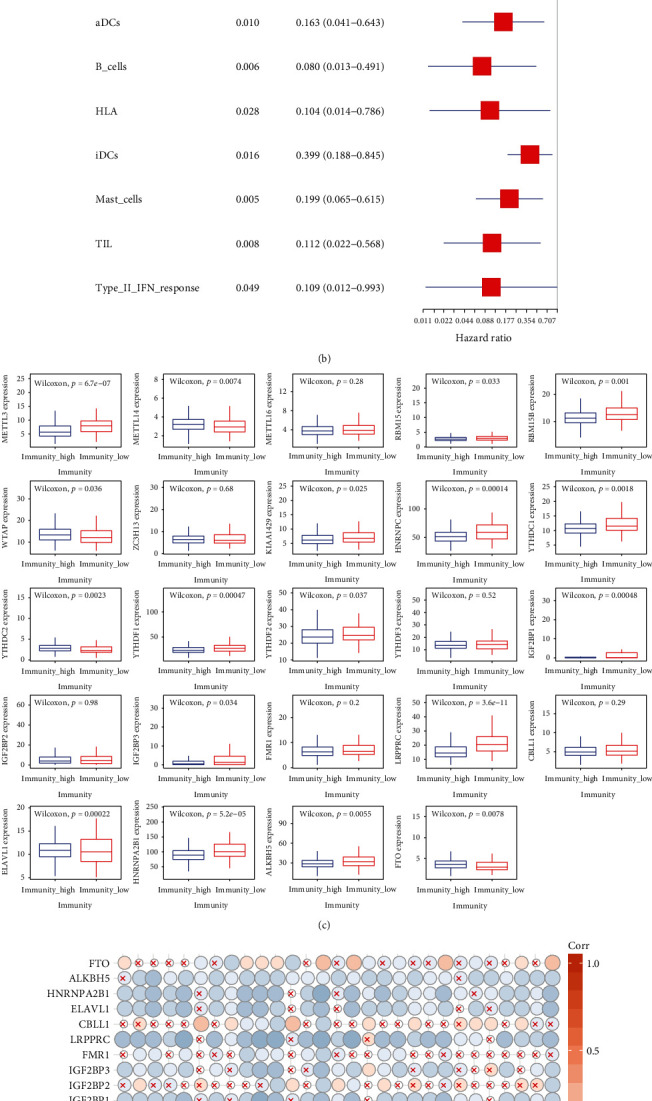
(a) Heatmap of ssGSEA scores of TCGA-LUAD. (b) Cox regression analysis for immune cells or pathways. Univariate Cox regression was used to calculate the hazard ratios (HR) and 95% confidence intervals (CI). (c) The expression of m6A methylation genes in immunity high and low group. (d) Correlations between 24 m6A methylation genes and 29 immune characters.

To understand the relationship between m6A modification and immune infiltration and response, the analysis started with evaluating the immune landscape. Based on the ssGSEA score of 29 immune cells or relative pathways and functions (immune characters), the high and low immune infiltration were defined. The ssGSEA scores of almost all the immune characters were lower in low immune infiltration LUAD (Figure 4(a)). The univariate Cox regression analysis was also applied, and the results showed that 7 immune characters were significantly correlated with OS. All of them are protective, with HR < 1 (Figure 4(b)). Comparing the expression of 24 m6A genes between the low and high immune infiltration groups, METTL3, RBM15, RBM15B, KIAA1429, HNRNPC, YTHDC1, YTHDF1, YTHDF2, IGF2BP1, IGF2BP3, LRPPRC, ELAVL1, HNRNPA2B1, and ALKBH5 were highly expressed in low immune infiltration group, while the low expression of METTL14, WTAP, YTHDC2, and FTO was observed in the same group (Figure 4(c)). Comprehensive relationships between m6A methylation genes and immune characters are calculated and displayed in Figure 4(d).

**Figure 5:**
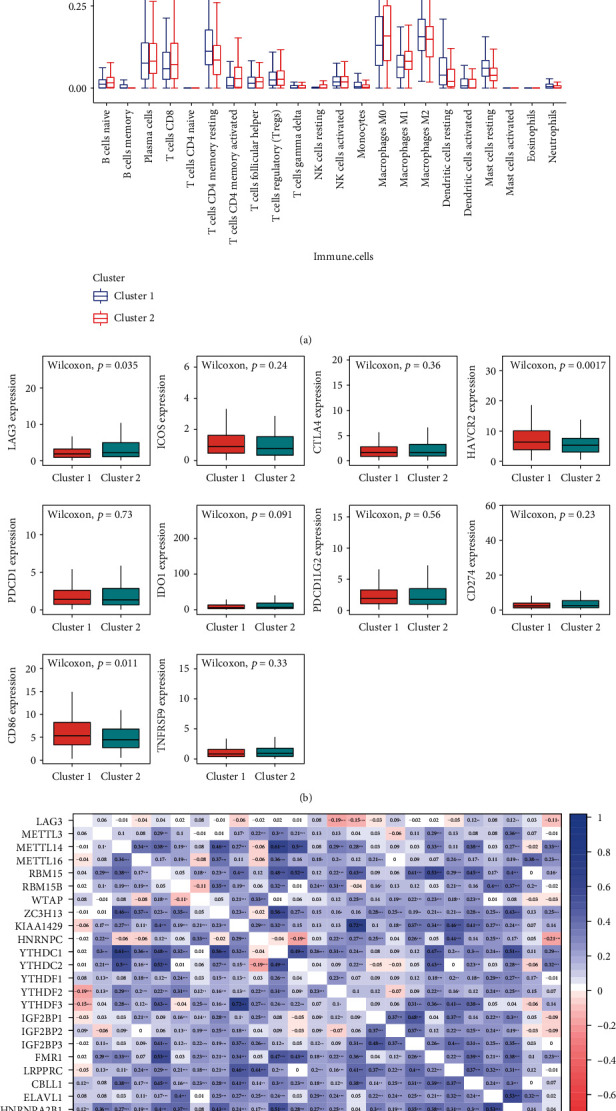
(a) The infiltration levels of 22 types of immune cells in clusters 1 and 2. (b) The expression of 10 immune-checkpoint-related genes in clusters 1 and 2. (c) The correlation analysis of LAG3 and m6A methylation genes.

Subsequently, we analyzed the fraction of 22 immune cell types between cluster 1 and cluster 2. As displayed in Figure 5(a), cluster 1 showed a higher infiltration level of memory B cells, CD4 naive T cells, CD4 memory-resting T cells, resting dendritic cells, and neutrophils, while the infiltration levels of CD4 memory-activated T cells, resting NK cells, M0 macrophages, M1 macrophages, and resting mast cells were higher in cluster 2. Since the significantly different infiltration of CD4 T cells was observed between the two clusters, we picked up 10 immune-checkpoint-related genes and compared their expression level in cluster 1 and cluster 2 (Figure 5(b)). Among them, the expression of LAG3 was higher in cluster 2, while HAVCR2 and CD86 had higher expression in cluster 1. Similar to PD-1 and CTLA-4, LAG3 plays an important role in Treg suppressive function and has a strong potential to become the target of anticancer drugs. Thus, we further explored the correlations between LAG3 and m6A methylation genes. Several “reader” m6A genes were significantly correlated with LAG3, including YTHDF2, YTHDF3, CBLL1, IGF2BP1, and HNRNPA2B1. Meanwhile, the abundance of an “eraser” gene, FTO, is also related to LAG3. Among them, IGF2BP2, CBLL1, and HNRNPA2B1 are positively correlated with the expression of LAG3, while YTHDF2, YTHDF3, and FTO are negatively correlated (Figure 5(c)).

### 3.4. Construction and Validation of the Prognostic Ability of m6A Methylation Signature

**Figure 6:**
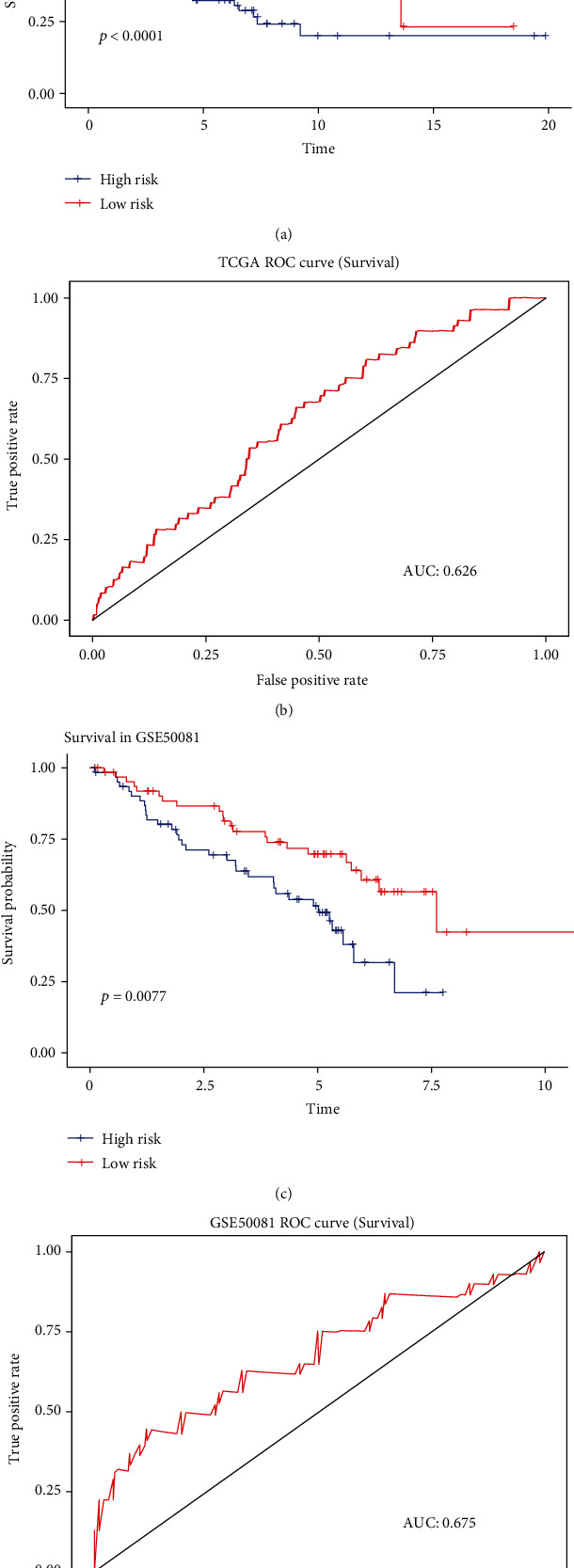
(a and c) ROC curves for the predictive value of risk score in TCGA (a) and GSE50081 (c) cohort. (b and d) Kaplan-Meier curves of OS for patients in TCGA (b) and GSE50081 (d) cohort with high risk and low risk.

We then explored the prognostic ability of these m6A methylation genes in patients with LUAD. The LASSO Cox regression analysis was conducted based on the expression value of above 24 m6A methylation genes from TCGA to better predict the survival rate of patients with LUAD. Eleven genes were selected by the LASSO algorithm, and the formula was generated as follows: Risk score = −0.033∗METTL3 + 0.0028∗KIAA1429 + 0.0087∗HNRNPC − 0.0053∗YTHDF1 − 0.0101∗YTHDF2 + 0.0413∗IGF2BP1 + 0.001∗IGF2BP2 + 0.0033∗IGFBP3 − 0.0165∗FMR1 − 0.0056∗LRPPRC + 0.0039∗HNRNPA2B1. All patients were designated to the high-risk group and the low-risk group according to the cut-off value that is set as the average value of the risk scores. As in Figures 6(a) and 6(b), the low-risk-score group has a better overall survival (*p* < 0.0001) than the high-risk-score group, and the AUC value is 0.701 in the TCGA cohort. The correction curve of this model is shown in Supplementary Figure 4 and the C-index value is 0.682. To further validate the prognostic ability, we used a validation cohort-GSE50081 and the AUC score of the validation cohort is 0.675 (Figure 6(c)). Meanwhile, a lower risk score is also related to better OS (Figure 6(d)).

**Figure 7:**
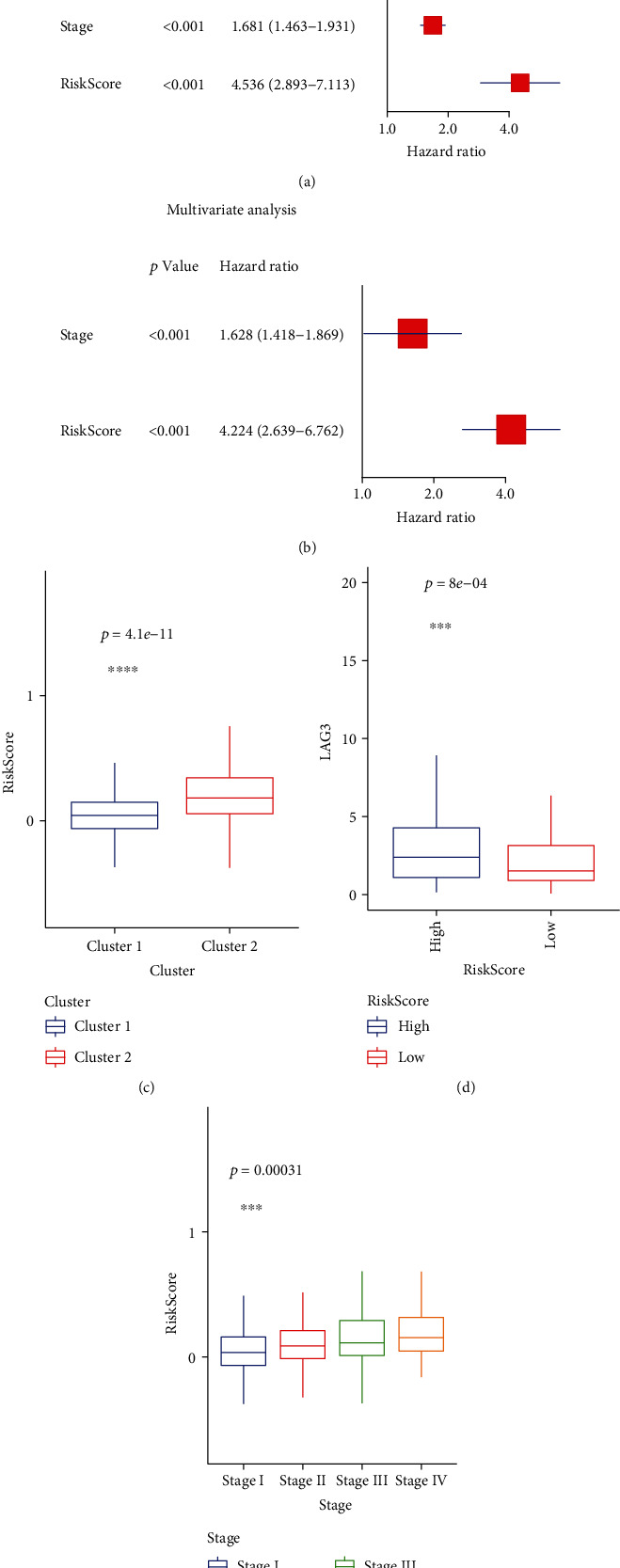
(a and b) Univariate Cox regression analysis for assessing the effects of clinical characters and risk score on the prognosis of LUAD. (b) Multivariate Cox regression analysis for assessing the effects of clinical characters and risk score on the prognosis of LUAD. (c) The distribution of risk score in clusters 1 and 2. (d) The expression of LAG3 in patients with high- and low-risk scores. (e) The distribution of risk score in different tumor stages.

Next, we analyzed the relationship between risk score and different tumor stages, clusters, and the expression level of m6A methylation genes and LAG3. Clearly in Figure 7(e), the risk score increases consistently with the tumor stages. Meanwhile, the lower risk score is correlated with cluster 1 and lower expression of LAG3 (Figures 7(c) and 7(d)). Besides, the expressions of WTAP, HNRNPC, YTHDF3, IGF2BP1, IGF2BP2, IGF2BP3, LRPPRC, ELAVL1, and HNRNPA2B1 were higher in patients with higher risk scores (Supplementary Figure 3). We also performed the univariate and multivariate Cox regression analyses to see if the risk score can independently predict the prognosis of patients with LUAD. In the univariate analysis (Figure 7(a)), only 2 of 4 factors, stage (*p* < 0.001) and risk score (*p* < 0.001), were associated with overall survival. Thus, they were included in the multivariate Cox regression analysis (Figure 7(b)), and these two factors remained significantly correlated with OS (both *p* < 0.001).

## 4. Discussion

As a dynamic and reversible process, m6A modification regulates mRNA by adding or deleting the methyl group on adenosine, as well as binding readers on modification sites, causing the subsequent biological effects. Understanding the function and the features of m6A regulators may extend our knowledge of the mechanism of tumorigenesis and provide new therapeutic methods or targets for cancer treatment.

Among the 18 genes that were differentially expressed between tumor and normal samples in our study, the expression of HNRNPC and HNRNPA2B1 was much higher than the others. These two genes belong to the hrnNP family. Heterogeneous nuclear ribonucleoproteins (hnRNPs) are commonly expressed in most human tissues. Several biological functions of hnRNPs were demonstrated, including mRNA genesis, DNA repair, telomere biogenesis, and regulation of gene expression. More and more evidence indicate that some hnRNPs are associated with the development and progression of tumors. For example, in breast cancer, knocking down hnRNPA2B1 leads to apoptosis of tumor cells, so hnRNPA2B1 is an oncogene in glioblastoma development and therefore may serve as a predictor of glioblastoma patient survival [41, 42]. From the perspective of signaling pathways, it is observed that hnRNPA2B1 plays an important role in STAT3 and ERK1/2 signaling transduction and activation [43]. Similar biological functions of hnRNPA2B1 are also observed in pancreatic cancer. It is reported that hnRNPA2B1 is highly expressed in pancreatic cancer and is related to higher expression of N-cadherin and vimentin, as well as lower expression of E-cadherin. Thus, hnRNPA2B1 may stimulate the epithelial-mesenchymal transition (EMT) [44]. With all these correlations between hnRNPA2B1 and cancer, this gene may have a high predictive value, as well as another heterogeneous nuclear ribonucleoprotein, hnRNPC. In breast cancer, the high expression of hnRNPC is also observed, and the inhibition of this gene leads to the accumulation of double-stranded RNA (dsRNA). Based on the computational inference and extensive experimental investigations, the cascade of interferon responses mediated by RIG-I may trigger this tumor-inhibitory effect, which means that its role in breast cancer might be controlling dsRNA and downstream interferon response [45]. In brief, hnRNPAB1 and hnRNPC are highly expressed in several cancers and have negative effects on OS, which is consistent with our result. The potential mechanism behind this phenomenon could be related to immune response.

N6-methyladenosine methylation is a complex biological progress and is not completely explored. With more and more m6A genes emerging and the lack of comprehensive understanding, it is hard to select m6A genes while exploring how N6-methyladenosine methylation may affect cancer or other diseases. Hence, another set of genes we would like to discuss is the eukaryotic translation initiation factors (EIFs), especially EIF3 gene family. Containing 13 subunits (EIF3A-EIF3M), EIF3 protein is a large complex that helps the binding of mRNA and ribosome [46]. Therefore, they belong to m6A reader genes and can control the downstream protein synthesis [47]. Many studies have shown that different EIF3 subunits may play different roles in different cancers. For example, EIF3A can promote the glycolysis in hepatocellular carcinoma by regulating the hypoxia-inducible factor 1-alpha [48]. EIF3B may function as an oncogenic protein that activates the PI3K/AKT/mTOR pathway in gastric cancer [49]. In addition, the translation facilitated by METTL16, which is important in the carcinogenesis of hepatocellular carcinoma, is mediated by the interaction between METTL16 and EIF3A and EIF3B [50]. EIF3C is the direct target of YTHDF1, and increased overall translational output of EIF3C by the regulation of YTHDF1 may facilitate the tumorigenesis and metastasis of ovarian cancer [51]. EIF3H subunits have been proved to physically and functionally interact with METTL3, which causes the translation of many oncogenic mRNA, including bromodomain-containing protein 4 which is modified by m6A in primary lung cancer [52]. The oncogenesis ability in lung adenocarcinoma of EIF3M has been proved by gain-and-loss function assay [53]. However, it is hard to decide whether genes of all these EIF3 subunits should be included in the analysis of m6A-related genes. On one hand, the potential role of EIF3 in LUAD is unignorable. On the other hand, it seems inappropriate to include all these EIF3 genes in the analysis when they function as a complex while having different expression pattern, especially EIF3L (Supplementary Figure 5). In addition, there is no significant difference between the survival rate of 2 clusters clustering with the data including EIF3 genes (*p* = 0.062), meaning that the subsequent analysis may not point out potential targets for clinical application, even though EIF3 is important in N6-methyladenosine methylation. Hence, EIF3 genes were not included in the main body of our work, but we still conducted some analysis to get a more comprehensive understanding of m6A in LUAD. Some worth-mentioning results are as follows. The expressions of EIFG3A-EIF3E and EIF3H-EIF3M are significantly different, and EIF3L is the only one that is downregulated in LUAD patients (Supplementary Figure 5). In the K-M analyses, worse survival is observed in patients with high expression of EIF3B, EIF3CL, EIF3D, EIF3H, and EIF3M (Supplementary Figure 7). Difference is found in the expression of EIF3A, EIF3B, EIF3D, EIF3F, EIF3H, EIF3J, and EIF3M in different tumor stages (Supplementary Figure 6). Only EIF3B and EIF3B show strong correlation with several genes sets in the 29 immune signatures (threshold: *p* < 0.05 and correlation coefficient > 0.3) (Supplementary Figure 8). The expression of EIF3A, EIF3B, EIF3G, EIF3H, EIF3J, and EIF3M was all lower in imunity_H group, except for EIF3G (Supplementary Figure 9). LAG3 shows negative correlation with EIF3 C, EIF3D, EIF3E, EIF3H, and EIF3L (Supplementary Figure 10). The univariate Cox regression analysis indicates that EIF3B, EIF3J, and EIF3M are the risk factors for the overall survival with hazard ratio = 1.012, 1.028, and 1.038, respectively (Supplementary Figure 11). All these results, though simple and superficial, provide a possibility that the expressions of EIF3 subunits are higher in LUAD patients and may directly or indirectly lead to a worse survival, partly through immune inhibition, but in a LAG3-independent way. Several studies have already reported the harmful effect of high expression of EIF3 subunits in lung cancer, including EIF3C [54], EIF3D [55], and EIF3H [52, 56]. In addition, a few studies focused on the immune regulation ability of EIF3, especially on the activation of T cells, including both cytotoxic T cells [57] and Treg cells [58]. In brief, EIF3 plays a really important role in LUAD, and how it may interact with other m6A components and immune regulation requires further investigation.

The result of GO enrichment analysis indicated that the DEGs between two m6A clusters were enriched in humoral immune responses. To our surprise, in addition to cancer cells, bacteria might also be one of the targets of such humoral immune response. Although lungs were considered sterile for a long time, the amount of microbiota colonized in lungs is beyond our expectations. A lot of studies have already demonstrated the existence of microbiota in lungs [59], and it is proved that the abundance of microbiota is different in lung cancer tissues and adjacent normal samples [60]. In addition, it seems like these microbiotas may participate in the pathogenesis of many lung diseases. For instance, after 210 bronchoscopic samples were analyzed, Laroumagne et al. identified several Gram-negative bacteria [61], which supported one of the biological processes that GO analysis enriched in our study. In vitro experiments demonstrated that Veillonella directly activates the ERK/PI3K pathway which represents the early event in lung cancer [62]. Except for the carcinogenesis signaling pathway, specific bacteria could promote cancer development and affect OS through immune response [63]. For instance, mycobacterium tuberculosis (TB) may contribute to carcinogenesis by causing chronic inflammation. The bacteria itself and its metabolites activate TLRs in immune and epithelial cells, triggering the start of inflammation and resulting in irreversible damage in normal cells [64, 65]. However, it is hard to precisely evaluate the difference in inflammation states between the two clusters based on transcriptome data, and we are also unable to acquire microbiota data in patents with LUAD to verify our hypothesis. Thus, prospective studies and multicenter clinical trials are required to provide further validation. Methods like using RNA-Seq data in TCGA to calculate the microbiota abundance in tumor tissue may also help to explain the role of microbiota in lung cancer [66].

Like programmed cell death 1 (PD-L1) and cytotoxic T lymphocyte antigen 4 (CTLA-4), LAG3 is an important checkpoint for immune responses. Continuous antigen stimulation could induce the expression of LAG3 on CD4+ and CD8+ T cells, which suppresses the function of these T cells and turns them into exhausted T cells [67]. Although the blockade of LAG3 does not result in the same effects as the PD-1 blockade [67], several studies have already focused on utilizing LAG3 as a target in cancer therapy [68]. In our study, we preliminarily analyzed the correlations between LAG3 and 24 m6A methylation genes in patients with LUAD. The result indicated that LAG3 is strongly correlated with several m6A regulators and could be one of the factors that affect the overall survival in cluster 1 and cluster 2, as well as in patients with high- and low-risk scores. Based on our educated guess, we hypothesized that the high expression of LAG3, caused by long-time anticancer immune response or microbiota-induced chronic inflammation, triggered a LAG3-mediated immunosuppressive microenvironment and subsequently leads to a worse prognosis. Interestingly, there is no difference in the expression of PD-1 between cluster 1 and cluster 2. Aoki et al. offered a possible reason since they identified a novel Hodgkin lymphoma-associated subset of T cells that highly express LAG3, and LAG3+ T cells are the mediator of immunosuppression [69]. Meanwhile, Jain et al. found that PD-1 is not overexpressed in leukemia antigen-specific T cells, but the overexpression of LAF3 and TIM3 was observed during the relapse [70]. Thus, considering the fact that PD-1 is not the only checkpoint in immune response, it is reasonable that no significant difference in PD-1 expression was observed between the two clusters in our study, since they might be clustered by other factors that are related to immunosuppress, including LAG3.

However, all our analyses were based on public data and were not validated in clinical cohorts. Conducting prospective research and validating the result from this study will be our next step, and we are looking forward to more clinical trials attempting to interpret the correlation between m6A and LUAD.

## 5. Conclusion

Our study evaluated the prognostic value, the correlations with LAG3, their role in immune infiltration, and the potential regulatory mechanisms of 24 m6A methylation genes in patients with LUAD. Two clusters of patients were identified via consensus clustering and were significantly different in their overall survival. Identified by GO analysis, the DEGs of these two groups were enriched in immune responses. A strong correlation between m6A methylation genes and immune infiltration was also observed and analyzed. Other than tumor stages, the risk score calculated by an eleven-gene-based signature is also a prognostic indicator for patients with LUAD. We hypothesized that the difference in overall survival between clusters 1 and 2, as well as patients with higher risk scores and lower risk scores, was partially caused by LAG3-mediated immunosuppression, which lead to a worse prognosis. Since LAG3 was significantly correlated with several m6A regulators, further research focusing on the potential mechanism behind these correlations and how m6A regulation could affect the expression of LAG3 on immune cells may provide promising targets for the immunotherapy of LUAD.

## Data Availability

The original datasets analyzed for this study can be found in The Cancer Genome Atlas program (https://portal.gdc.cancer.gov/) and Gene Expression Omnibus with the accession code GSE50081 (https://www.ncbi.nlm.nih.gov/geo/). All data generated during this study are included in this article.

## Supplementary Materials

Supplementary Materials Supplementary Figure 1: Kaplan-Meier curves indicating the effects of m6A methylation genes on the overall survival in patients with LUAD. Supplementary Figure 2: genes that belonged to DEGs in clusters 1 and 2 and were listed in ImmPort and InnateDB with the threshold set on *p* value < 0.05 and |*logFC*| > 0.5. Supplementary Figure 3: the expression of m6A methylation genes in patients with high and low risk score. Supplementary Figure 4: the correction curve of the prediction model. Supplementary Figure 5: the expression of EIF3 subunits in LUAD patents and controls. Supplementary Figure 6: the expression of EIF3 subunits in different stages. Supplementary Figure 7: Kaplan-Meier curves indicating the effects of EIF3 subunits expression on the overall survival in patients with LUAD. Supplementary Figure 8: correlations between EIF3 subunits and 29 immune characters. Supplementary Figure 9: the expression of EIF3 subunits in immunity-high and -low group. Supplementary Figure 10: the expression of EIF3 subunits in immunity-high and -low group. Supplementary Figure 11: univariate Cox regression analysis for assessing the effects of EIF3 subunits on the prognosis of LUAD. Supplementary Table 1: genes that belonged to DEGs in clusters 1 and 2 and were listed in ImmPort and InnateDB with the threshold set on *p* value < 0.05 and |*logFC*| > 0.5.
