# Supplementary material for: Effects of N6-Methyladenosine Regulators on LAG3 and Immune Infiltrates in Lung Adenocarcinoma
Source: Dis Markers. 2022 Aug 23;2022:1829528. doi: 10.1155/2022/1829528 (PMC9427291; doi:10.1155/2022/1829528)
Supplement: Supplementary Materials — Supplementary Figure 1: Kaplan-Meier curves indicating the effects of m6A methylation genes on the overall survival in patients with LUAD. Supplementary Figure 2: genes that belonged to DEGs in clusters 1 and 2 and were listed in ImmPort and InnateDB with the threshold set on p value < 0.05 and |logFC| > 0.5. Supplementary Figure 3: the expression of m6A methylation genes in patients with high and low risk score. Supplementary Figure 4: the correction curve of the prediction model. Supplementary Figure 5: the expression of EIF3 subunits in LUAD patents and controls. Supplementary Figure 6: the expression of EIF3 subunits in different stages. Supplementary Figure 7: Kaplan-Meier curves indicating the effects of EIF3 subunits expression on the overall survival in patients with LUAD. Supplementary Figure 8: correlations between EIF3 subunits and 29 immune characters. Supplementary Figure 9: the expression of EIF3 subunits in immunity-high and -low group. Supplementary Figure 10: the expression of EIF3 subunits in immunity-high and -low group. Supplementary Figure 11: univariate Cox regression analysis for assessing the effects of EIF3 subunits on the prognosis of LUAD. Supplementary Table 1: genes that belonged to DEGs in clusters 1 and 2 and were listed in ImmPort and InnateDB with the threshold set on p value < 0.05 and |logFC| > 0.5. [file 1829528.f1.docx]

## Supplementary Figures


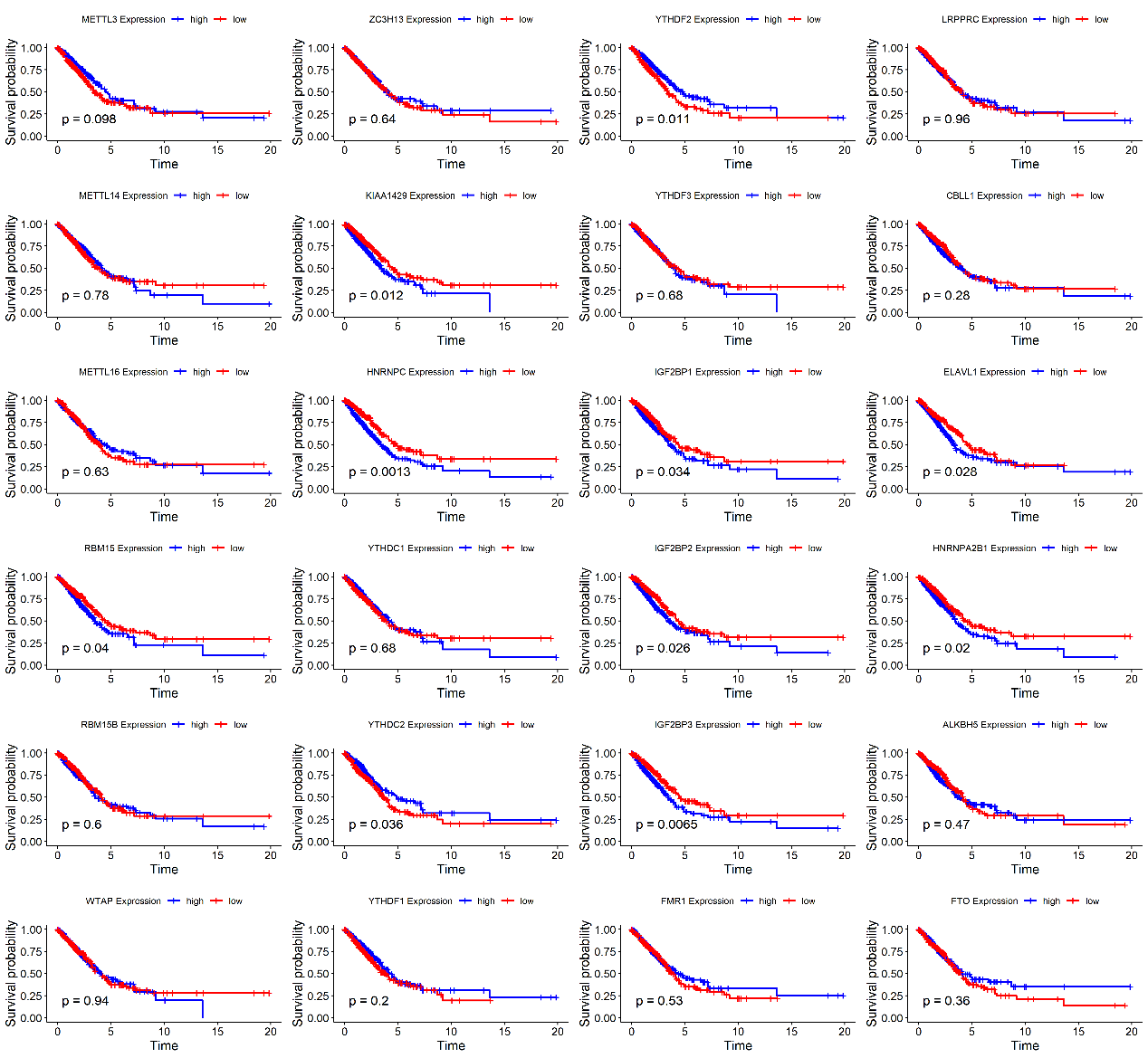


**Supplementary Figure 1.** Kaplan-Meier curves indicating the effects of m6A-related genes on the overall survival in patients with LUAD.


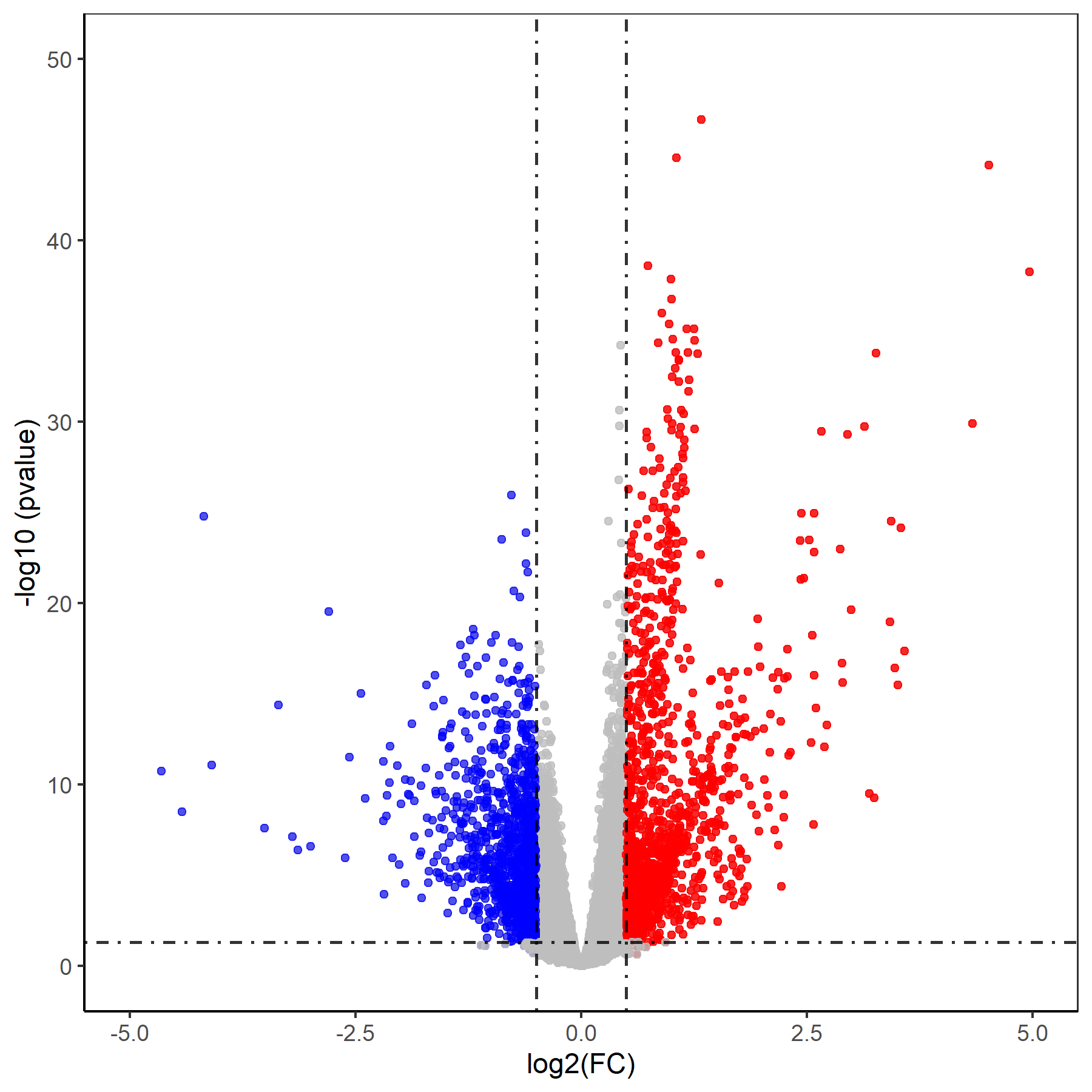


**Supplementary Figure 2.** Genes that belonged to DEGs in cluster 1 & 2 and were listed in ImmPort and InnateDB. With the threshold set on P-value <0.05 and | logFC | > 0.5. The details of these DEGs were list in supplementary table 1.


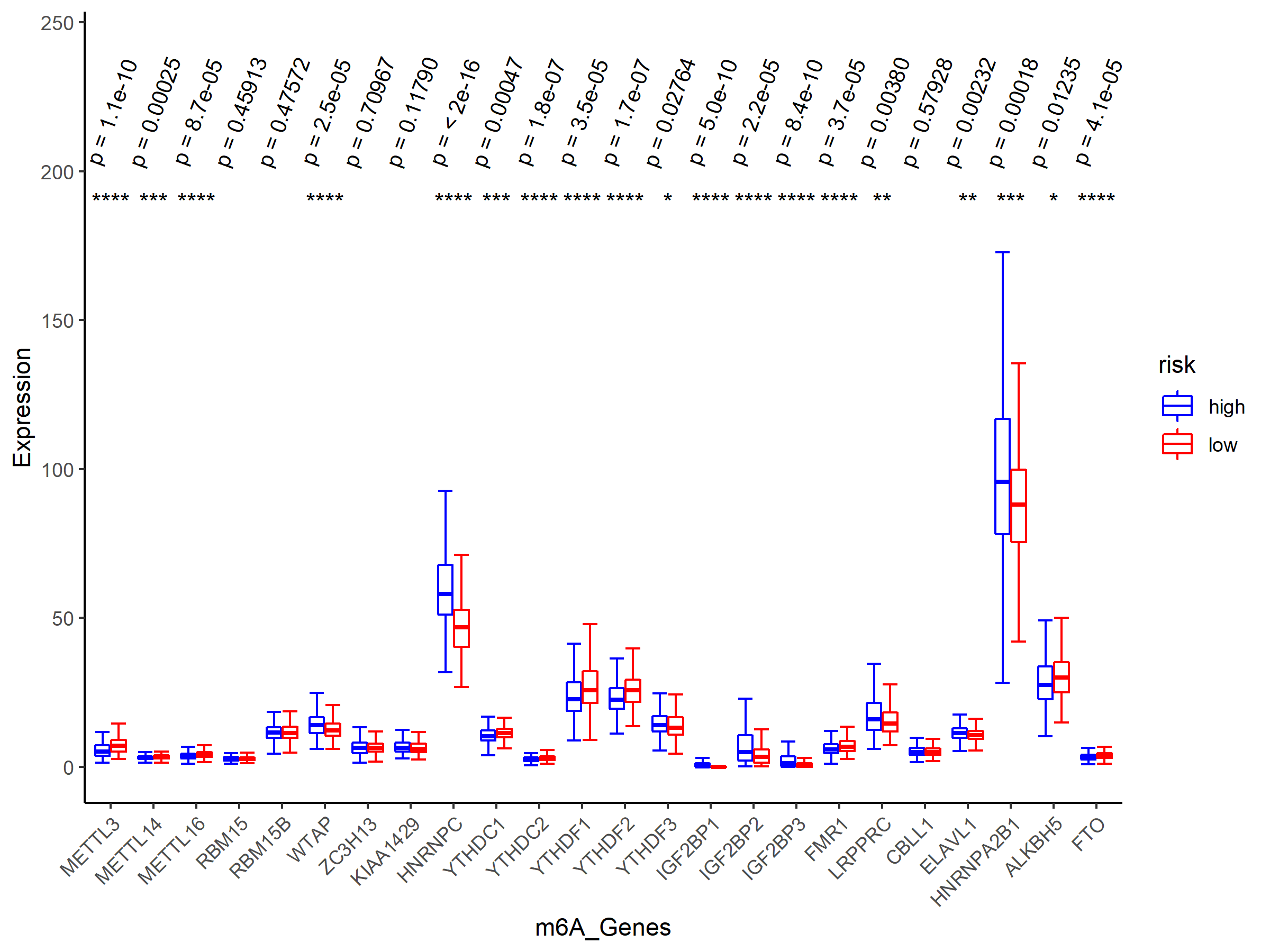


**Supplementary Figure 3.** The expression of m6A-related genes in patients with high- and low risk-score.


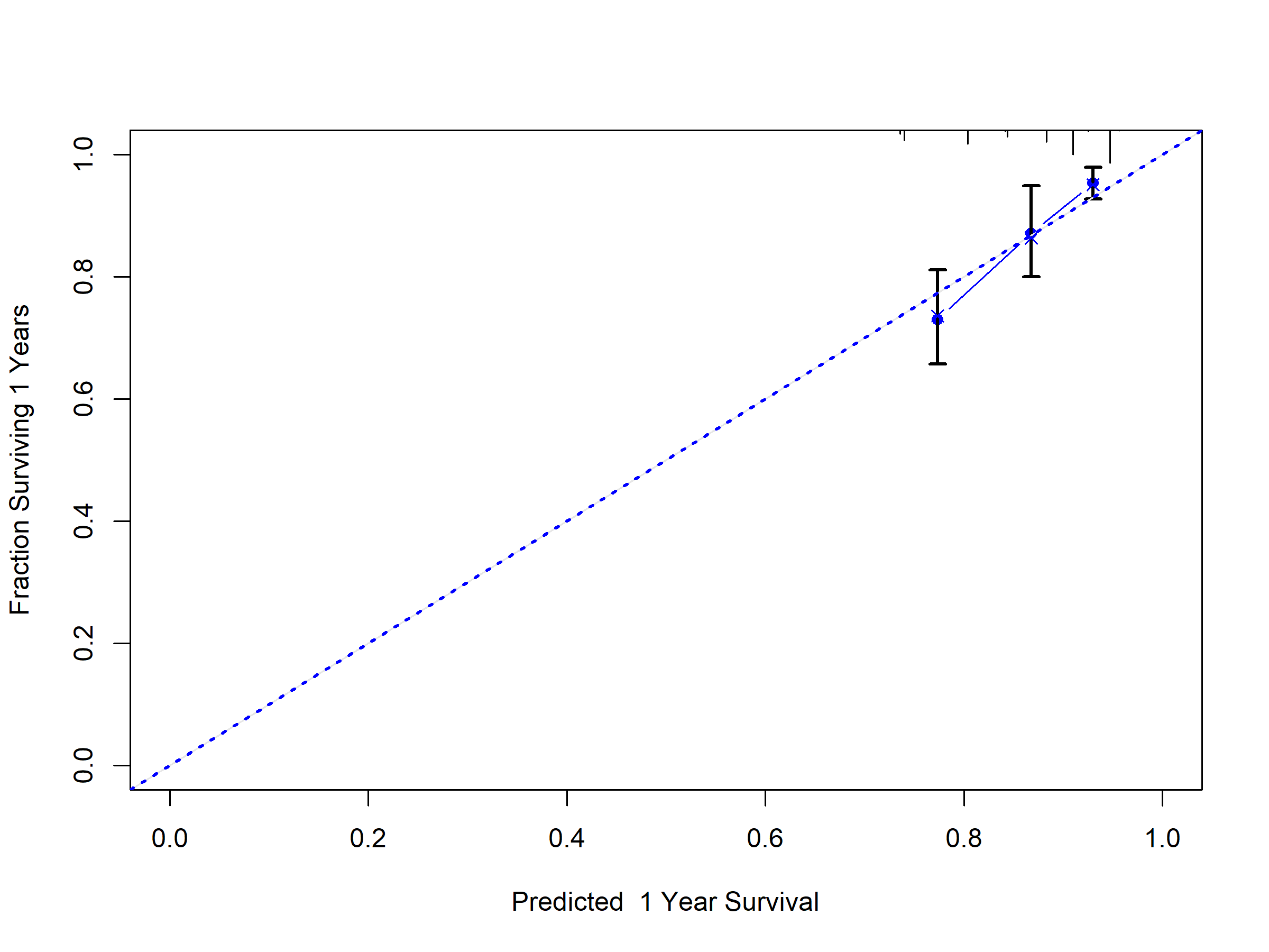


**Supplementary Figure 4.** The correction curve of the prediction model.


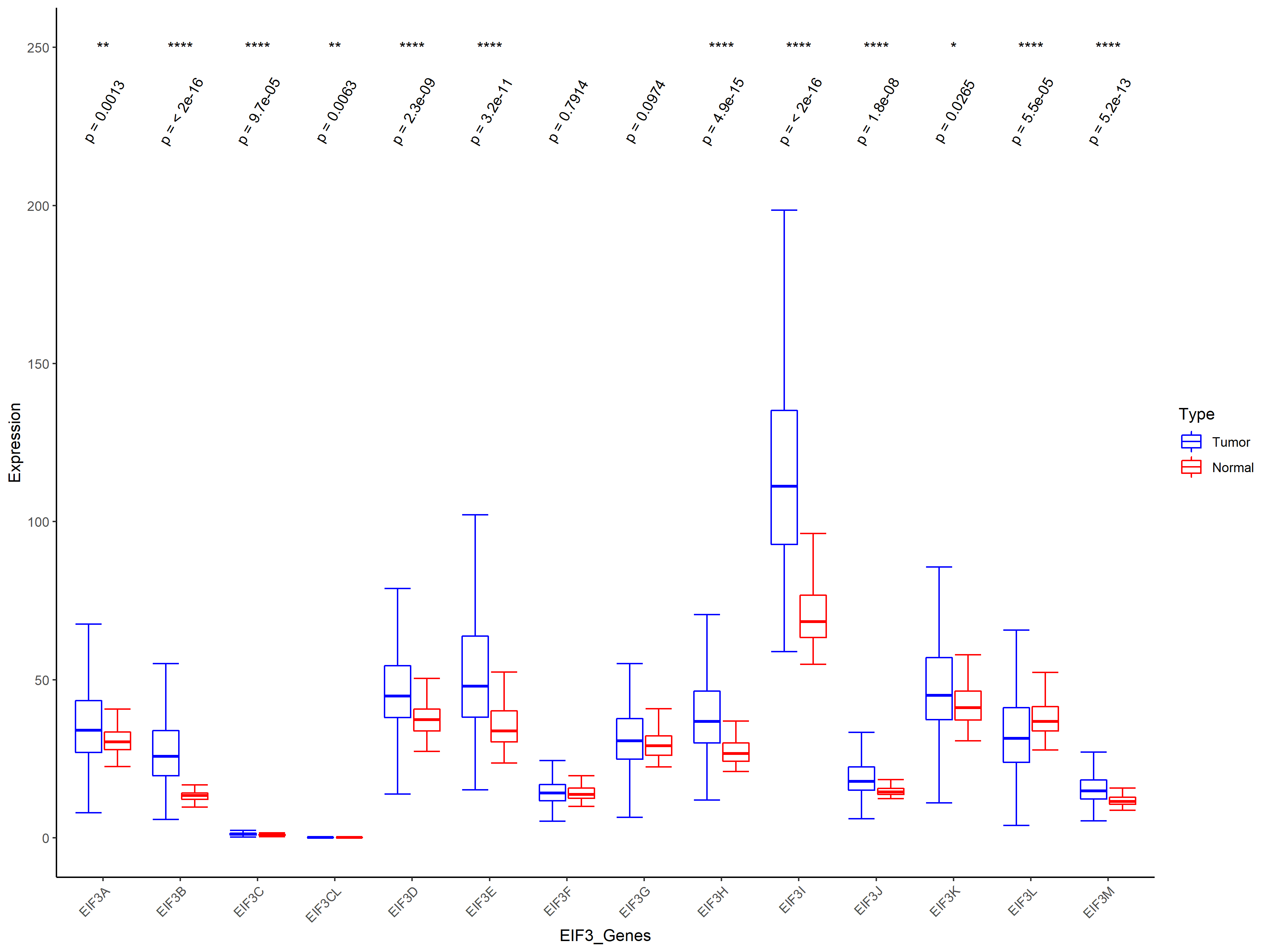


**Supplementary Figure 5.** The expression of EIF3 subunits in LUAD patents and controls.


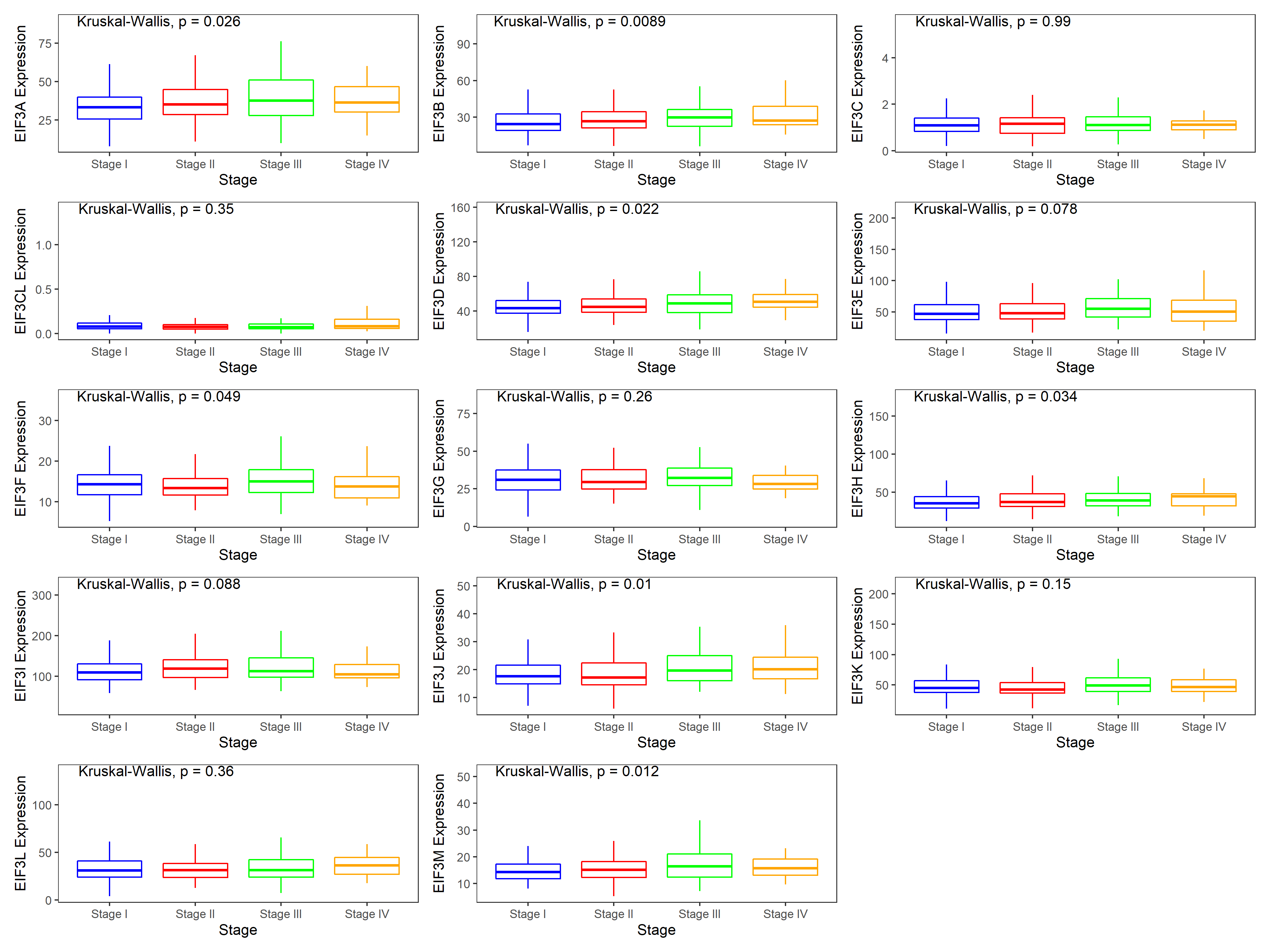


**Supplementary Figure 6.** The expression of EIF3 subunits in different stages.


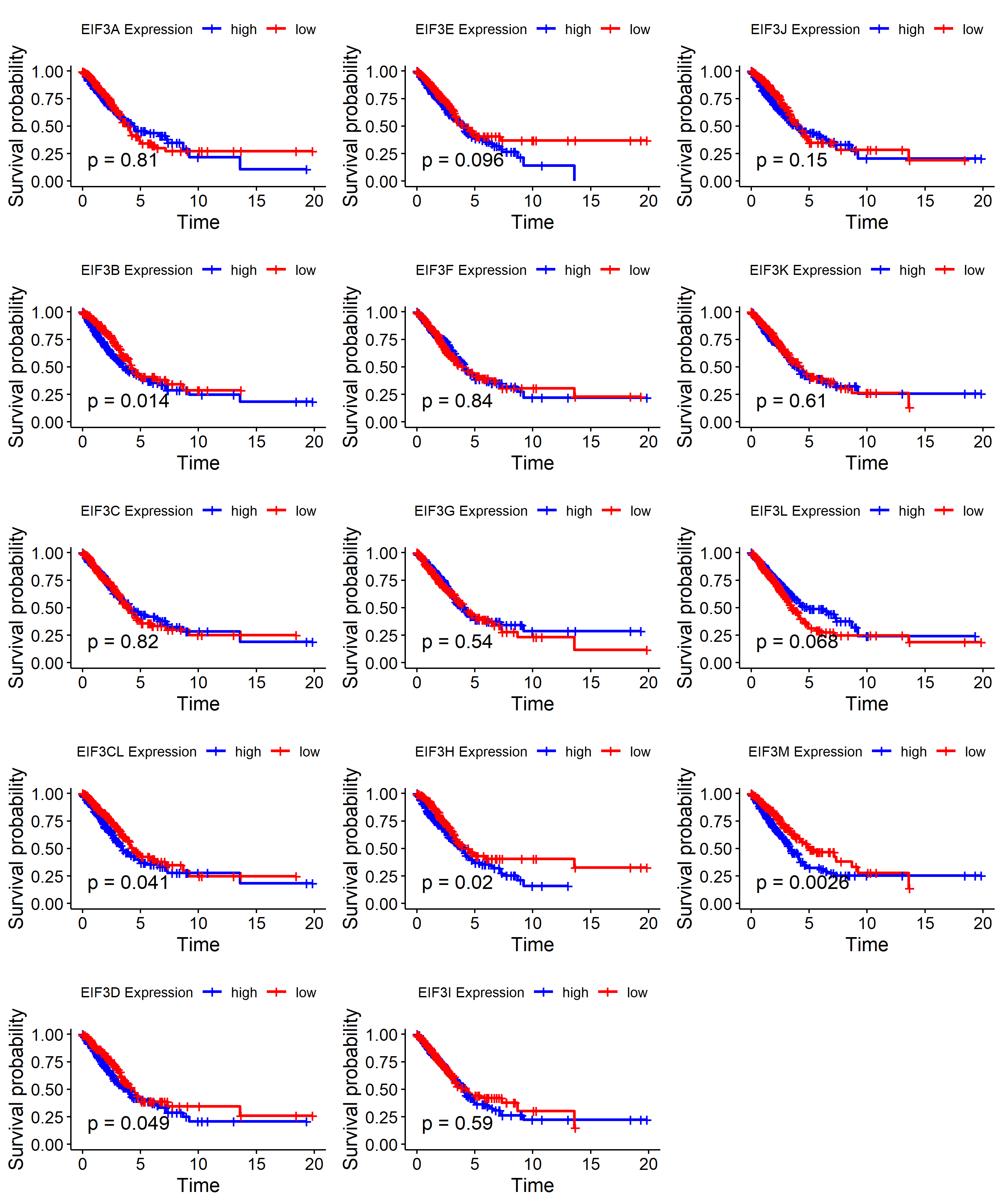


**Supplementary Figure 7.** Kaplan-Meier curves indicating the effects of EIF3 subunits expression on the overall survival in patients with LUAD.


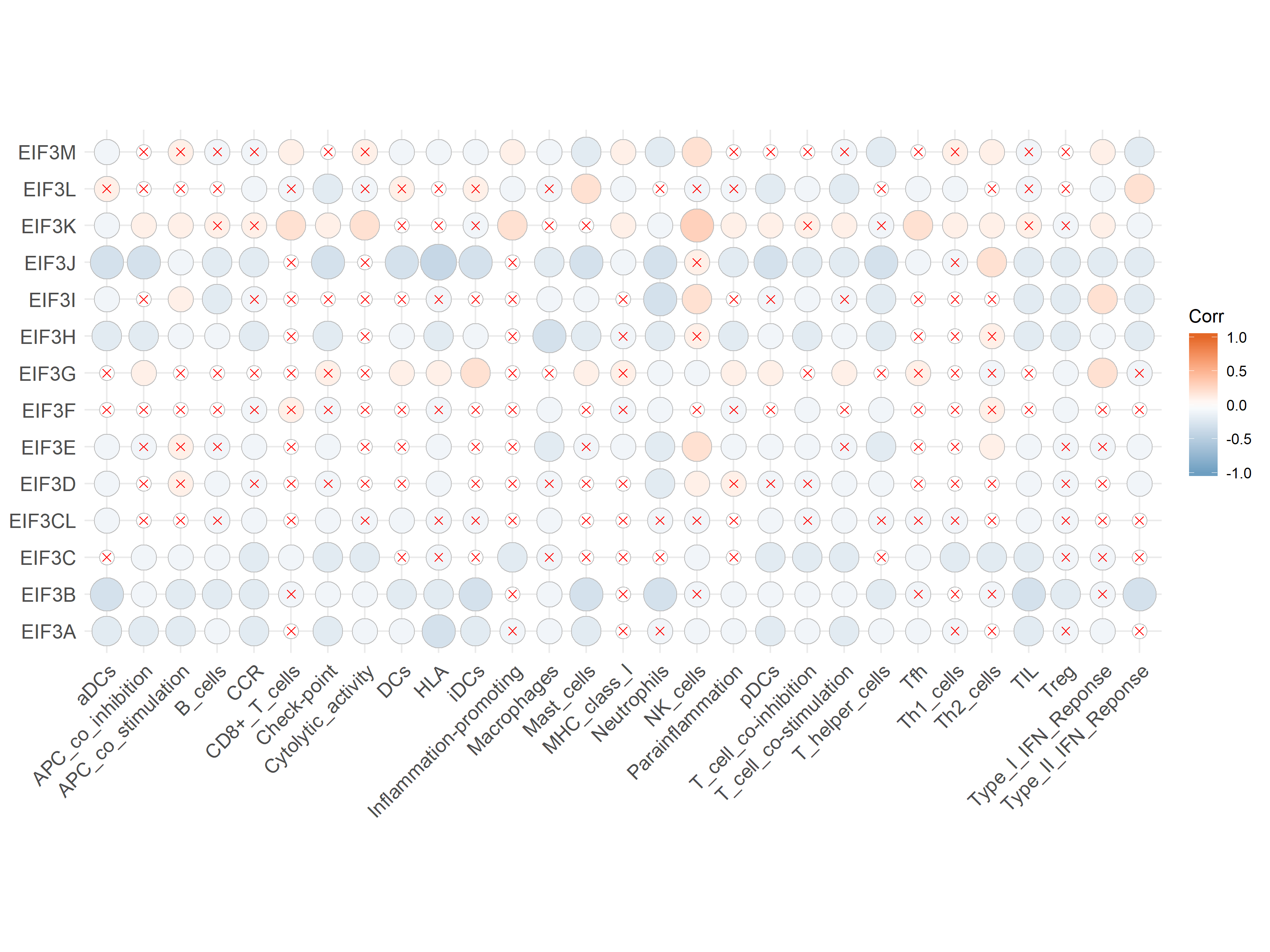


**Supplementary Figure 8.** Correlations between EIF3 subunits and 29 immune characters.


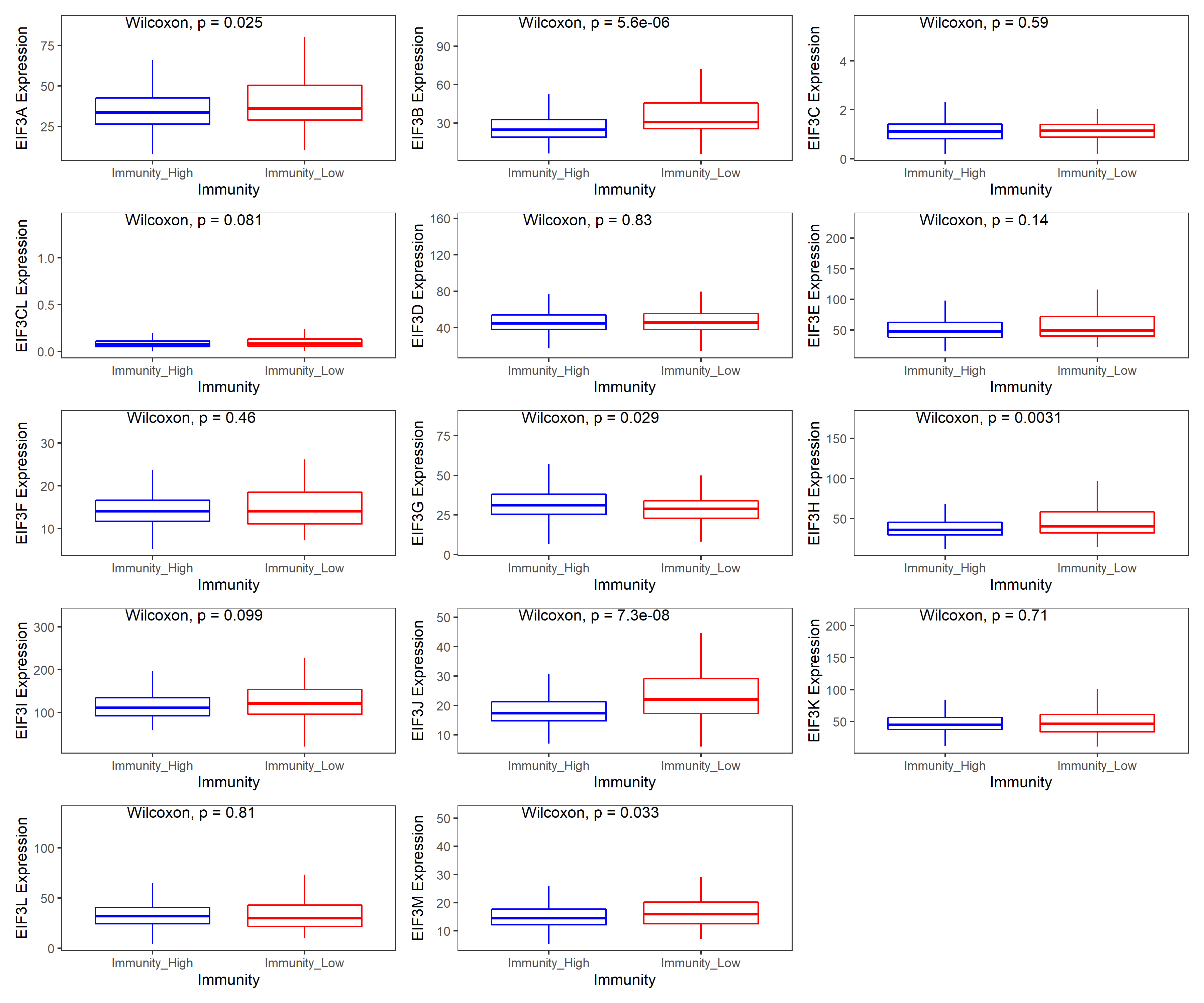


**Supplementary Figure 9.** The expression of EIF3 subunits in immunity-high and -low group.


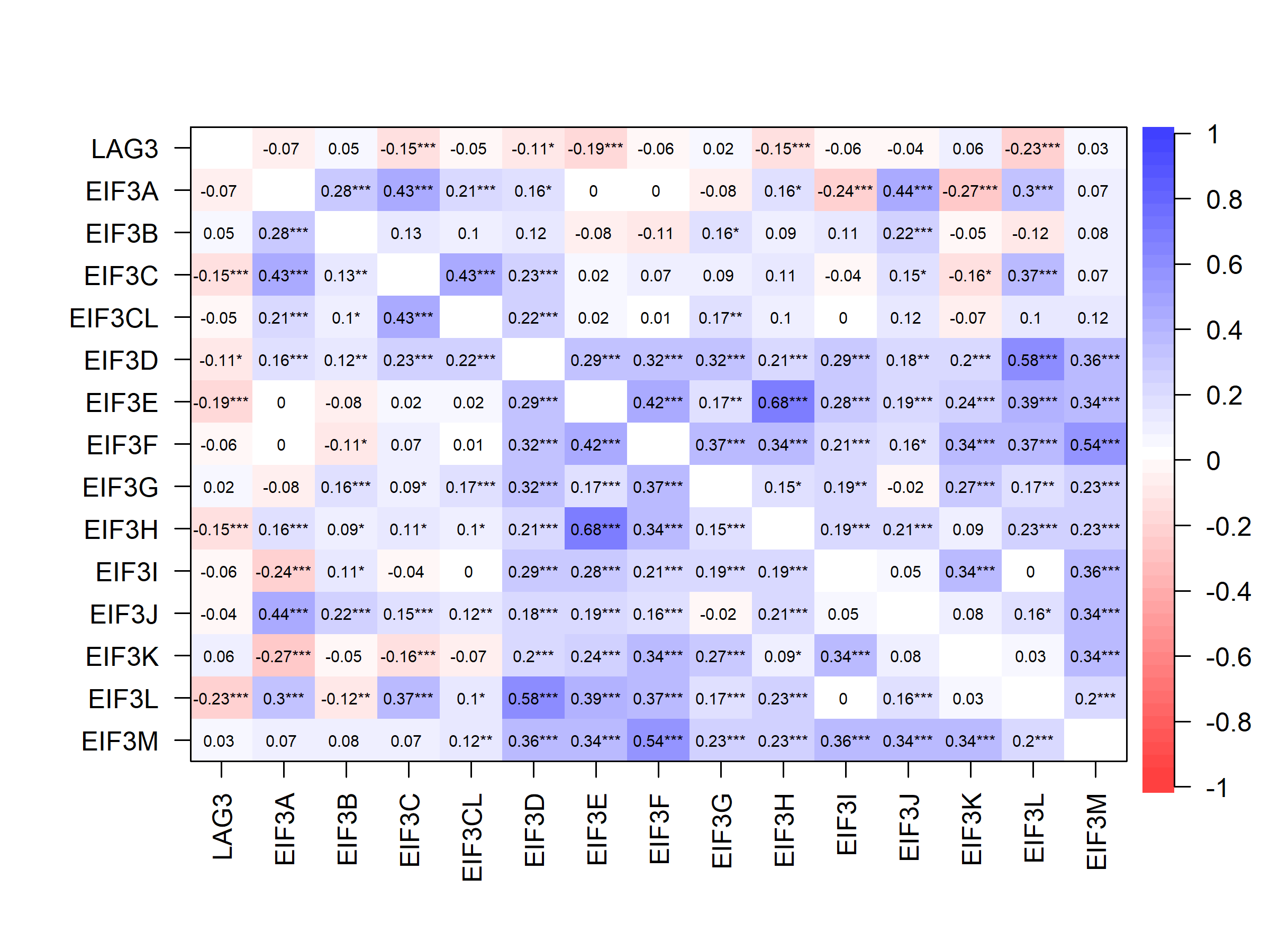


**Supplementary Figure 10.** The expression of EIF3 subunits in immunity-high and -low group.


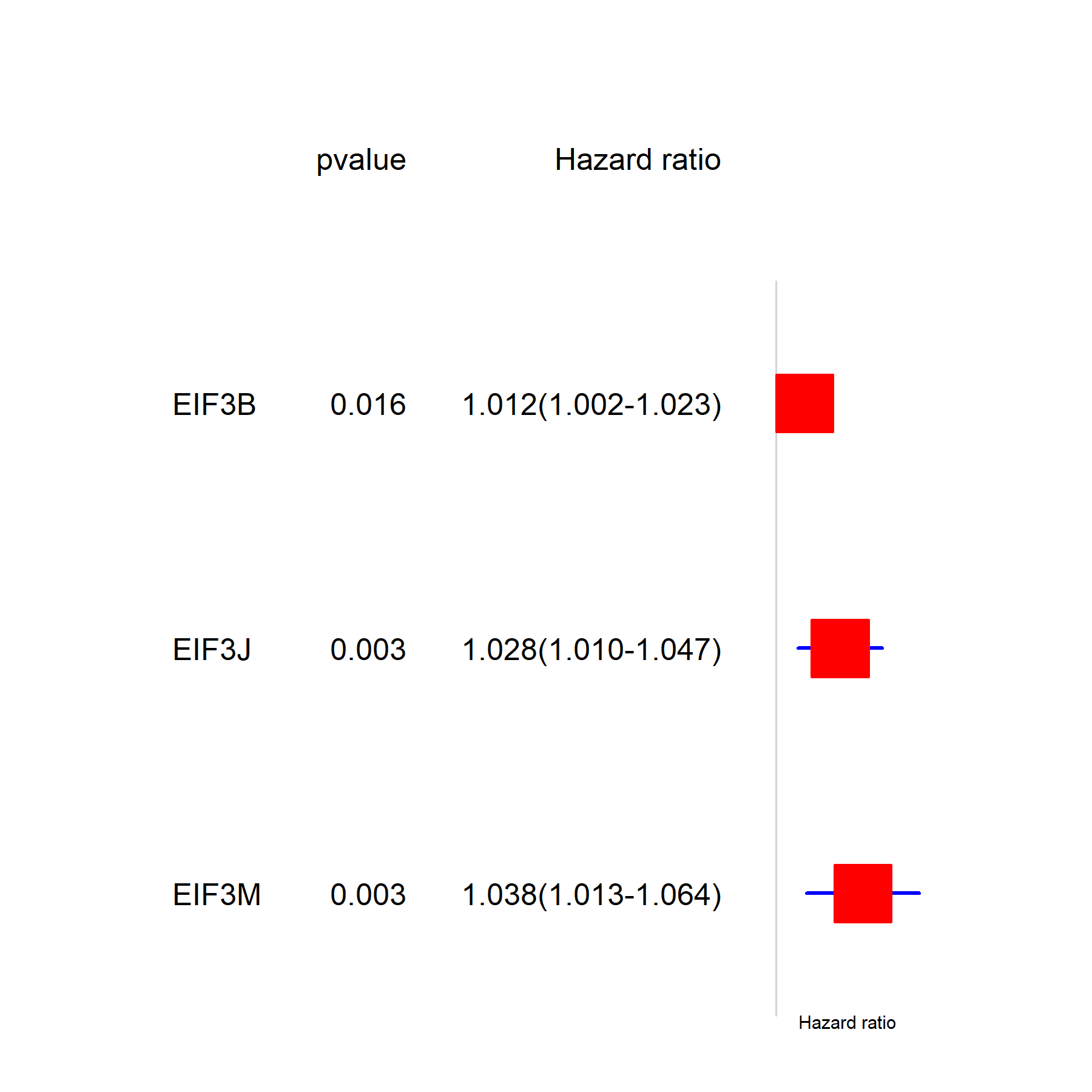


**Supplementary Figure 11.** Univariate Cox regression analysis for assessing the effects of EIF3 subunits on the prognosis of LUAD.

## Supplementary Tables

| Differentially Expressed Genes | | | |  |  |  |
| --- | --- | --- | --- | --- | --- | --- |
| gene | logFC | logCPM | LR | PValue | FDR | change |
| DEFA5 | 6.727307 | 4.905474 | 213.9327 | 1.91E-48 | 1.16E-44 | UP |
| KIR2DL1 | 3.266728 | 3.161776 | 150.1313 | 1.62E-34 | 1.41E-31 | UP |
| VTN | 3.135715 | 4.787345 | 131.5991 | 1.83E-30 | 8.78E-28 | UP |
| APOA1 | 2.950823 | 3.439101 | 129.6503 | 4.89E-30 | 2.02E-27 | UP |
| SKP2 | 0.949966 | 4.755246 | 116.9768 | 2.90E-27 | 8.53E-25 | UP |
| FCGRT | -0.77522 | 7.347079 | 114.2703 | 1.14E-26 | 3.05E-24 | DOWN |
| HMGB2 | 0.79211 | 6.940249 | 111.0878 | 5.66E-26 | 1.39E-23 | UP |
| CD81 | -0.61001 | 8.040229 | 104.791 | 1.36E-24 | 2.67E-22 | DOWN |
| SCG2 | 2.52374 | 5.245762 | 103.0747 | 3.23E-24 | 6.00E-22 | UP |
| CRH | 2.867645 | 2.967772 | 100.7399 | 1.05E-23 | 1.75E-21 | UP |
| VGF | 2.578144 | 3.980731 | 100.0275 | 1.50E-23 | 2.47E-21 | UP |
| PLK1 | 0.942877 | 4.78159 | 99.66442 | 1.81E-23 | 2.94E-21 | UP |
| TNFSF12 | -0.61348 | 5.83271 | 97.01968 | 6.86E-23 | 1.04E-20 | DOWN |
| E2F1 | 0.909401 | 5.361108 | 96.74295 | 7.89E-23 | 1.18E-20 | UP |
| PRKDC | 0.688198 | 5.938041 | 96.42832 | 9.25E-23 | 1.34E-20 | UP |
| TNFSF13 | -0.67699 | 5.345725 | 88.63661 | 4.74E-21 | 5.58E-19 | DOWN |
| WDR62 | 1.047863 | 3.590899 | 86.98906 | 1.09E-20 | 1.21E-18 | UP |
| DEFA6 | 2.988432 | 2.981914 | 85.45952 | 2.36E-20 | 2.48E-18 | UP |
| IL33 | -1.19409 | 5.137131 | 80.57813 | 2.79E-19 | 2.61E-17 | DOWN |
| LY86 | -0.94583 | 5.141078 | 79.14461 | 5.77E-19 | 5.26E-17 | DOWN |
| CTSH | -1.2308 | 8.511877 | 77.88131 | 1.09E-18 | 9.77E-17 | DOWN |
| FGF3 | 2.281438 | 2.906079 | 75.55999 | 3.54E-18 | 2.92E-16 | UP |
| CD1C | -1.31348 | 4.418553 | 71.66549 | 2.55E-17 | 1.89E-15 | DOWN |
| CTSD | -0.68501 | 10.8336 | 71.34638 | 3.00E-17 | 2.18E-15 | DOWN |
| TPT1 | -0.70426 | 10.65224 | 70.37434 | 4.91E-17 | 3.42E-15 | DOWN |
| CD63 | -0.56855 | 10.3622 | 68.29631 | 1.41E-16 | 8.93E-15 | DOWN |
| CCNA2 | 0.855222 | 5.10341 | 67.07815 | 2.61E-16 | 1.58E-14 | UP |
| AGER | -1.7112 | 7.061408 | 66.61317 | 3.30E-16 | 1.96E-14 | DOWN |
| ALB | 3.505102 | 7.040609 | 66.59465 | 3.33E-16 | 1.97E-14 | UP |
| PLA2G2A | -2.43651 | 5.289833 | 64.51261 | 9.59E-16 | 5.30E-14 | DOWN |
| ACKR1 | -1.18114 | 4.858902 | 63.90397 | 1.31E-15 | 7.10E-14 | DOWN |
| IFNGR1 | -0.56924 | 7.446129 | 63.45721 | 1.64E-15 | 8.78E-14 | DOWN |
| CXCL16 | -0.58371 | 7.566411 | 62.57957 | 2.56E-15 | 1.32E-13 | DOWN |
| WFDC12 | -3.35518 | 4.077774 | 61.55814 | 4.30E-15 | 2.13E-13 | DOWN |
| SFTPD | -1.63187 | 9.207693 | 61.37354 | 4.72E-15 | 2.32E-13 | DOWN |
| C7 | -1.31804 | 6.116551 | 59.97158 | 9.62E-15 | 4.59E-13 | DOWN |
| LST1 | -0.87772 | 5.148197 | 59.51658 | 1.21E-14 | 5.69E-13 | DOWN |
| TPSB2 | -1.16614 | 5.476728 | 59.12834 | 1.48E-14 | 6.78E-13 | DOWN |
| CHGA | 2.722756 | 4.036555 | 56.64245 | 5.23E-14 | 2.23E-12 | UP |
| CX3CR1 | -1.09602 | 3.56935 | 56.37835 | 5.98E-14 | 2.52E-12 | DOWN |
| TREM2 | -0.91771 | 6.482364 | 55.46474 | 9.52E-14 | 3.86E-12 | DOWN |
| TMEM173 | -0.61725 | 6.808884 | 55.42737 | 9.70E-14 | 3.93E-12 | DOWN |
| GNRH2 | 1.830642 | 3.245139 | 54.14827 | 1.86E-13 | 7.24E-12 | UP |
| AGT | 1.434476 | 5.895417 | 52.89224 | 3.52E-13 | 1.30E-11 | UP |
| PTGDS | -0.96539 | 6.759668 | 52.0395 | 5.44E-13 | 1.95E-11 | DOWN |
| CAT | -0.54609 | 6.767467 | 52.02152 | 5.49E-13 | 1.96E-11 | DOWN |
| GMFG | -0.63485 | 5.934677 | 50.45124 | 1.22E-12 | 4.16E-11 | DOWN |
| S1PR1 | -0.6261 | 5.083558 | 49.21259 | 2.30E-12 | 7.46E-11 | DOWN |
| HSPA2 | 0.859497 | 4.00939 | 48.59297 | 3.15E-12 | 9.95E-11 | UP |
| CD1E | -1.16886 | 3.771074 | 47.95466 | 4.36E-12 | 1.34E-10 | DOWN |
| BIRC5 | 0.799616 | 5.56536 | 47.57283 | 5.30E-12 | 1.62E-10 | UP |
| SCGB3A1 | -2.1868 | 10.90649 | 47.52705 | 5.43E-12 | 1.65E-10 | DOWN |
| CCRL2 | -0.73268 | 3.998385 | 46.91934 | 7.40E-12 | 2.20E-10 | DOWN |
| NPY | -4.09288 | 5.022787 | 46.60558 | 8.68E-12 | 2.56E-10 | DOWN |
| CD1A | -1.47237 | 4.594164 | 46.26339 | 1.03E-11 | 3.01E-10 | DOWN |
| ELN | -0.90988 | 6.067415 | 46.11053 | 1.12E-11 | 3.21E-10 | DOWN |
| FAM3D | -1.72181 | 4.461215 | 45.83355 | 1.29E-11 | 3.66E-10 | DOWN |
| CTSG | -1.17596 | 3.398467 | 45.76129 | 1.34E-11 | 3.78E-10 | DOWN |
| EGFR | 0.829366 | 5.951391 | 45.39817 | 1.61E-11 | 4.48E-10 | UP |
| FCN3 | -1.11945 | 4.588139 | 44.2671 | 2.86E-11 | 7.61E-10 | DOWN |
| C4BPA | -1.38827 | 8.595099 | 44.14421 | 3.05E-11 | 8.03E-10 | DOWN |
| CCL17 | -1.27541 | 4.806778 | 43.3764 | 4.52E-11 | 1.15E-09 | DOWN |
| TYROBP | -0.71108 | 8.334625 | 43.23147 | 4.86E-11 | 1.23E-09 | DOWN |
| TRAIP | 0.631187 | 3.686459 | 43.05865 | 5.31E-11 | 1.33E-09 | UP |
| AGTR2 | -1.89035 | 4.061318 | 42.68857 | 6.42E-11 | 1.57E-09 | DOWN |
| EDNRB | -0.81264 | 4.20113 | 42.31398 | 7.77E-11 | 1.85E-09 | DOWN |
| LYZ | -1.05226 | 10.15016 | 41.99907 | 9.13E-11 | 2.14E-09 | DOWN |
| TNFAIP8L2 | -0.60831 | 4.775772 | 41.20579 | 1.37E-10 | 3.09E-09 | DOWN |
| GRP | 1.423236 | 4.249522 | 40.55663 | 1.91E-10 | 4.17E-09 | UP |
| CCL23 | -0.92273 | 3.613082 | 39.89882 | 2.67E-10 | 5.70E-09 | DOWN |
| TAC1 | 3.190143 | 3.957258 | 39.52885 | 3.23E-10 | 6.81E-09 | UP |
| C3 | -0.879 | 9.04885 | 39.5137 | 3.26E-10 | 6.85E-09 | DOWN |
| CST4 | -1.91353 | 3.712544 | 39.37393 | 3.50E-10 | 7.28E-09 | DOWN |
| CD300LF | -0.72957 | 4.477097 | 39.20996 | 3.81E-10 | 7.87E-09 | DOWN |
| SFTPA2 | -1.50322 | 12.21336 | 38.69599 | 4.95E-10 | 9.98E-09 | DOWN |
| GCG | 3.247675 | 3.402301 | 38.44807 | 5.62E-10 | 1.12E-08 | UP |
| DES | -1.0448 | 4.590717 | 37.9561 | 7.24E-10 | 1.42E-08 | DOWN |
| DUSP1 | -0.76553 | 9.196476 | 37.94673 | 7.27E-10 | 1.42E-08 | DOWN |
| GDF10 | -1.11605 | 3.558115 | 37.85778 | 7.61E-10 | 1.48E-08 | DOWN |
| CTSE | -1.40915 | 8.734662 | 37.54176 | 8.95E-10 | 1.71E-08 | DOWN |
| DCN | -0.66221 | 6.878333 | 37.25491 | 1.04E-09 | 1.95E-08 | DOWN |
| IFRD1 | 0.519643 | 4.834305 | 37.21614 | 1.06E-09 | 1.98E-08 | UP |
| LGALS3 | -0.52786 | 9.13858 | 36.38339 | 1.62E-09 | 2.92E-08 | DOWN |
| C6 | -1.30905 | 3.294874 | 36.04955 | 1.92E-09 | 3.39E-08 | DOWN |
| TNFRSF10C | -0.61063 | 4.268849 | 35.65564 | 2.35E-09 | 4.08E-08 | DOWN |
| DMBT1 | -1.49835 | 6.657555 | 35.40242 | 2.68E-09 | 4.60E-08 | DOWN |
| HLA-DPB1 | -0.65057 | 9.149302 | 35.3224 | 2.79E-09 | 4.77E-08 | DOWN |
| COLEC12 | -0.72287 | 4.838445 | 34.99367 | 3.31E-09 | 5.58E-08 | DOWN |
| CD74 | -0.63889 | 12.09155 | 34.90915 | 3.45E-09 | 5.79E-08 | DOWN |
| CD37 | -0.58172 | 5.266241 | 34.78942 | 3.67E-09 | 6.12E-08 | DOWN |
| HLA-DRA | -0.66965 | 12.08154 | 34.53793 | 4.18E-09 | 6.84E-08 | DOWN |
| CHGB | 1.940742 | 5.499721 | 34.28639 | 4.76E-09 | 7.69E-08 | UP |
| GDF6 | 1.351553 | 2.945056 | 34.15035 | 5.10E-09 | 8.21E-08 | UP |
| RFTN1 | -0.5705 | 5.953921 | 33.90117 | 5.80E-09 | 9.22E-08 | DOWN |
| DEFB1 | -1.54235 | 6.127532 | 33.75553 | 6.25E-09 | 9.82E-08 | DOWN |
| CD4 | -0.50581 | 6.562052 | 33.67987 | 6.50E-09 | 1.02E-07 | DOWN |
| HP | -1.70877 | 6.411655 | 33.56307 | 6.90E-09 | 1.08E-07 | DOWN |
| ELANE | -1.38905 | 3.146511 | 33.19983 | 8.32E-09 | 1.27E-07 | DOWN |
| PTGFR | -1.20541 | 3.256096 | 32.85375 | 9.94E-09 | 1.50E-07 | DOWN |
| PGC | -2.19153 | 11.5215 | 32.7784 | 1.03E-08 | 1.55E-07 | DOWN |
| IRF8 | -0.55931 | 4.676346 | 32.29636 | 1.32E-08 | 1.94E-07 | DOWN |
| PYCARD | -0.55541 | 5.782853 | 32.16281 | 1.42E-08 | 2.07E-07 | DOWN |
| SEMA4A | -0.50788 | 5.121246 | 32.12733 | 1.44E-08 | 2.10E-07 | DOWN |
| NR3C2 | -0.58976 | 4.015133 | 31.86624 | 1.65E-08 | 2.37E-07 | DOWN |
| LTB | -0.69089 | 5.687694 | 31.38609 | 2.11E-08 | 2.96E-07 | DOWN |
| LRRK2 | -1.00071 | 5.112374 | 31.37709 | 2.12E-08 | 2.98E-07 | DOWN |
| NPR1 | -0.73652 | 4.061149 | 31.20711 | 2.32E-08 | 3.22E-07 | DOWN |
| HLA-DRB1 | -0.65963 | 11.23884 | 31.20185 | 2.33E-08 | 3.23E-07 | DOWN |
| BTK | -0.51068 | 4.159573 | 31.10101 | 2.45E-08 | 3.38E-07 | DOWN |
| DUOX1 | -0.88477 | 4.622635 | 31.08531 | 2.47E-08 | 3.40E-07 | DOWN |
| SCARF1 | -0.5746 | 4.403445 | 30.63217 | 3.12E-08 | 4.19E-07 | DOWN |
| HLA-DMB | -0.5755 | 6.379958 | 30.21202 | 3.87E-08 | 5.08E-07 | DOWN |
| FOS | -0.72778 | 8.552753 | 30.02295 | 4.27E-08 | 5.55E-07 | DOWN |
| NLRP7 | 1.135648 | 2.913416 | 29.86833 | 4.62E-08 | 5.95E-07 | UP |
| CD1B | -0.93654 | 3.316386 | 29.55629 | 5.43E-08 | 6.87E-07 | DOWN |
| LTBP2 | -0.56477 | 6.632926 | 29.25852 | 6.33E-08 | 7.84E-07 | DOWN |
| SLC22A3 | -0.80851 | 5.429835 | 29.07483 | 6.96E-08 | 8.53E-07 | DOWN |
| GATA6 | -0.66548 | 4.320321 | 28.97972 | 7.31E-08 | 8.90E-07 | DOWN |
| IL34 | -0.73395 | 3.822864 | 28.7904 | 8.06E-08 | 9.70E-07 | DOWN |
| C8B | -1.34511 | 3.371957 | 28.69955 | 8.45E-08 | 1.01E-06 | DOWN |
| CD79B | -0.72971 | 4.43074 | 28.58633 | 8.96E-08 | 1.07E-06 | DOWN |
| CFP | -0.78757 | 3.285751 | 28.48353 | 9.45E-08 | 1.12E-06 | DOWN |
| HLA-DMA | -0.53575 | 7.982284 | 27.80536 | 1.34E-07 | 1.53E-06 | DOWN |
| TLR2 | -0.58875 | 5.599846 | 27.71268 | 1.41E-07 | 1.59E-06 | DOWN |
| FANCC | 0.516173 | 3.662255 | 27.68666 | 1.43E-07 | 1.61E-06 | UP |
| IL17RE | -0.56404 | 4.70911 | 27.54335 | 1.54E-07 | 1.73E-06 | DOWN |
| STC1 | 0.781055 | 5.275891 | 27.41355 | 1.64E-07 | 1.84E-06 | UP |
| RNASE7 | 1.16488 | 2.919702 | 27.40054 | 1.65E-07 | 1.85E-06 | UP |
| NPPA | 0.992299 | 2.972655 | 27.05214 | 1.98E-07 | 2.17E-06 | UP |
| PACSIN1 | 0.77862 | 3.170599 | 26.29615 | 2.93E-07 | 3.09E-06 | UP |
| KIR3DL2 | 1.100024 | 2.893449 | 26.06889 | 3.29E-07 | 3.43E-06 | UP |
| OPRD1 | 0.952807 | 2.954573 | 25.9287 | 3.54E-07 | 3.66E-06 | UP |
| OGN | -1.01631 | 3.876726 | 25.90231 | 3.59E-07 | 3.71E-06 | DOWN |
| ANGPT1 | -0.66232 | 3.669596 | 25.88174 | 3.63E-07 | 3.74E-06 | DOWN |
| VIPR1 | -0.63394 | 3.883038 | 25.74085 | 3.90E-07 | 3.97E-06 | DOWN |
| FGR | -0.50524 | 4.888871 | 25.623 | 4.15E-07 | 4.20E-06 | DOWN |
| REG1A | -3.13684 | 4.582516 | 25.60102 | 4.20E-07 | 4.24E-06 | DOWN |
| CALCRL | -0.50128 | 4.657302 | 25.53813 | 4.34E-07 | 4.37E-06 | DOWN |
| STC2 | 0.699982 | 4.241324 | 25.36102 | 4.75E-07 | 4.75E-06 | UP |
| FPR1 | -0.56426 | 4.678359 | 25.02546 | 5.66E-07 | 5.52E-06 | DOWN |
| HLA-DPA1 | -0.56908 | 8.430133 | 24.92567 | 5.96E-07 | 5.80E-06 | DOWN |
| HLA-DOA | -0.59338 | 6.111123 | 24.72457 | 6.61E-07 | 6.37E-06 | DOWN |
| TRIM45 | 0.500587 | 3.616421 | 24.47753 | 7.52E-07 | 7.15E-06 | UP |
| MSR1 | -0.59072 | 5.129032 | 24.34467 | 8.05E-07 | 7.59E-06 | DOWN |
| CFTR | -0.86589 | 3.937548 | 24.23923 | 8.51E-07 | 7.94E-06 | DOWN |
| CD22 | -0.69813 | 3.679613 | 24.17204 | 8.81E-07 | 8.20E-06 | DOWN |
| P2RY14 | -0.58654 | 3.540051 | 24.11328 | 9.08E-07 | 8.40E-06 | DOWN |
| AGRP | -0.84679 | 3.196159 | 24.07246 | 9.28E-07 | 8.55E-06 | DOWN |
| SCTR | -0.94389 | 4.913598 | 23.95433 | 9.86E-07 | 9.04E-06 | DOWN |
| FCN1 | -0.67588 | 4.027057 | 23.81446 | 1.06E-06 | 9.66E-06 | DOWN |
| CGB5 | -2.6129 | 3.828078 | 23.68981 | 1.13E-06 | 1.02E-05 | DOWN |
| AZU1 | -1.13042 | 3.129776 | 23.54194 | 1.22E-06 | 1.09E-05 | DOWN |
| CCL14 | -0.88057 | 3.069817 | 23.45044 | 1.28E-06 | 1.14E-05 | DOWN |
| TSC22D3 | -0.52635 | 7.508284 | 23.44806 | 1.28E-06 | 1.14E-05 | DOWN |
| ORM1 | -1.21242 | 5.977953 | 23.36015 | 1.34E-06 | 1.19E-05 | DOWN |
| ZBTB16 | -0.91476 | 3.327835 | 23.18461 | 1.47E-06 | 1.29E-05 | DOWN |
| C8A | -1.50779 | 3.14918 | 22.93788 | 1.67E-06 | 1.45E-05 | DOWN |
| AQP3 | -0.77389 | 9.425411 | 22.86545 | 1.74E-06 | 1.50E-05 | DOWN |
| GDF15 | -0.75246 | 7.411398 | 22.71008 | 1.88E-06 | 1.62E-05 | DOWN |
| NR4A3 | -0.80269 | 3.913015 | 22.66468 | 1.93E-06 | 1.65E-05 | DOWN |
| RETN | -0.96151 | 4.412404 | 22.66445 | 1.93E-06 | 1.65E-05 | DOWN |
| GKN1 | -1.62962 | 2.940376 | 22.66022 | 1.93E-06 | 1.65E-05 | DOWN |
| CR2 | -0.87711 | 4.050998 | 22.62488 | 1.97E-06 | 1.68E-05 | DOWN |
| IL11 | 0.756804 | 3.225483 | 22.35581 | 2.27E-06 | 1.90E-05 | UP |
| CCL19 | -0.76547 | 6.908577 | 21.68741 | 3.21E-06 | 2.60E-05 | DOWN |
| CHIT1 | -0.95419 | 5.534273 | 21.66499 | 3.25E-06 | 2.62E-05 | DOWN |
| BMP7 | 0.89775 | 3.630303 | 21.64925 | 3.27E-06 | 2.64E-05 | UP |
| MMP7 | -0.90381 | 7.456614 | 21.46604 | 3.60E-06 | 2.87E-05 | DOWN |
| MIA | -1.45119 | 3.387035 | 21.32627 | 3.87E-06 | 3.06E-05 | DOWN |
| C8G | 0.773172 | 3.454734 | 21.29826 | 3.93E-06 | 3.10E-05 | UP |
| CXCL11 | 0.756078 | 4.589193 | 21.19071 | 4.16E-06 | 3.26E-05 | UP |
| HRG | 1.019878 | 2.838955 | 20.97696 | 4.65E-06 | 3.59E-05 | UP |
| SPINK5 | -1.05256 | 4.971205 | 20.9641 | 4.68E-06 | 3.61E-05 | DOWN |
| SIGLEC7 | -0.51095 | 3.616558 | 20.89515 | 4.85E-06 | 3.72E-05 | DOWN |
| VEGFC | 0.6008 | 4.453793 | 20.86363 | 4.93E-06 | 3.78E-05 | UP |
| HLA-DRB5 | -0.66342 | 9.877332 | 20.82504 | 5.03E-06 | 3.84E-05 | DOWN |
| TLR10 | -0.72469 | 3.425281 | 20.80568 | 5.08E-06 | 3.87E-05 | DOWN |
| HLA-DQB1 | -0.58599 | 8.007556 | 20.80293 | 5.09E-06 | 3.87E-05 | DOWN |
| FGFR2 | -0.58213 | 4.269887 | 20.69825 | 5.38E-06 | 4.06E-05 | DOWN |
| PMAIP1 | 0.563462 | 5.41073 | 20.6853 | 5.41E-06 | 4.09E-05 | UP |
| S100A6 | -0.53939 | 12.55038 | 20.56898 | 5.75E-06 | 4.32E-05 | DOWN |
| VAV3 | 0.591123 | 3.752818 | 19.96608 | 7.88E-06 | 5.67E-05 | UP |
| IL17RD | 0.617342 | 3.331855 | 19.92435 | 8.06E-06 | 5.78E-05 | UP |
| BMP2 | -0.61574 | 5.015795 | 19.74694 | 8.84E-06 | 6.28E-05 | DOWN |
| IDO1 | 0.709638 | 5.90895 | 19.73804 | 8.88E-06 | 6.30E-05 | UP |
| PYY | 0.88791 | 2.931172 | 19.42309 | 1.05E-05 | 7.26E-05 | UP |
| HSPA6 | 0.638512 | 4.997493 | 19.021 | 1.29E-05 | 8.77E-05 | UP |
| LGALS4 | -1.56317 | 6.459932 | 18.84507 | 1.42E-05 | 9.51E-05 | DOWN |
| GNLY | -0.87906 | 5.144589 | 18.82113 | 1.44E-05 | 9.62E-05 | DOWN |
| TRIM36 | 0.676331 | 3.263042 | 18.73891 | 1.50E-05 | 0.0001 | UP |
| SEMA5B | 0.673273 | 3.119635 | 18.55687 | 1.65E-05 | 0.000108 | UP |
| GNRHR | 0.861292 | 2.862982 | 18.34819 | 1.84E-05 | 0.000119 | UP |
| TFR2 | 0.633213 | 3.267487 | 18.31021 | 1.88E-05 | 0.000121 | UP |
| ADRB2 | -0.50451 | 3.64732 | 18.28005 | 1.91E-05 | 0.000123 | DOWN |
| HOXA9 | 0.936926 | 3.005324 | 18.25287 | 1.93E-05 | 0.000124 | UP |
| INHBE | 0.852222 | 2.993898 | 18.00924 | 2.20E-05 | 0.000139 | UP |
| FGFR3 | -0.71162 | 5.220217 | 18.00192 | 2.21E-05 | 0.00014 | DOWN |
| INS | -1.69367 | 2.986982 | 17.73172 | 2.54E-05 | 0.000158 | DOWN |
| AR | -0.69811 | 3.264259 | 17.71046 | 2.57E-05 | 0.000159 | DOWN |
| PTH1R | -0.60122 | 3.346628 | 17.66471 | 2.63E-05 | 0.000163 | DOWN |
| SOCS2 | -0.55675 | 4.248925 | 17.54845 | 2.80E-05 | 0.000171 | DOWN |
| EPGN | 0.964012 | 2.990311 | 17.50315 | 2.87E-05 | 0.000175 | UP |
| NLRP11 | 1.031343 | 2.938008 | 17.49589 | 2.88E-05 | 0.000175 | UP |
| HLA-DQA1 | -0.51052 | 7.24588 | 17.359 | 3.09E-05 | 0.000187 | DOWN |
| NR4A1 | -0.63174 | 6.090596 | 17.35773 | 3.10E-05 | 0.000187 | DOWN |
| CCL13 | -0.66809 | 6.028701 | 17.09943 | 3.55E-05 | 0.000211 | DOWN |
| KCNJ8 | -0.53954 | 4.798556 | 17.07884 | 3.59E-05 | 0.000213 | DOWN |
| CGA | 1.841242 | 4.930034 | 16.84362 | 4.06E-05 | 0.000238 | UP |
| CDKN2A | 0.749177 | 4.866141 | 16.6148 | 4.58E-05 | 0.000264 | UP |
| CXCR1 | -0.75804 | 3.032856 | 16.41466 | 5.09E-05 | 0.00029 | DOWN |
| PPBP | -0.99107 | 3.839317 | 16.12681 | 5.92E-05 | 0.000331 | DOWN |
| CXCL5 | 0.854702 | 5.259833 | 15.9204 | 6.61E-05 | 0.000365 | UP |
| MARCO | -0.67182 | 6.77693 | 15.80399 | 7.03E-05 | 0.000386 | DOWN |
| TDGF1 | 0.806197 | 2.861499 | 15.74917 | 7.23E-05 | 0.000396 | UP |
| F11 | -0.81709 | 3.056197 | 15.39397 | 8.73E-05 | 0.000467 | DOWN |
| CXCR2 | -0.65637 | 3.129857 | 15.29985 | 9.17E-05 | 0.000488 | DOWN |
| BMX | -0.91155 | 3.28453 | 15.07792 | 0.000103 | 0.000543 | DOWN |
| CMA1 | -0.78787 | 2.945987 | 15.04948 | 0.000105 | 0.000549 | DOWN |
| TRH | 0.798528 | 2.854635 | 14.97786 | 0.000109 | 0.000569 | UP |
| IL5RA | -0.77107 | 3.017356 | 14.94967 | 0.00011 | 0.000577 | DOWN |
| OSM | -0.53574 | 4.151488 | 14.89687 | 0.000114 | 0.000589 | DOWN |
| FOXA2 | -0.5935 | 5.59588 | 14.54393 | 0.000137 | 0.000695 | DOWN |
| SEMA3B | -0.55642 | 4.967731 | 14.30627 | 0.000155 | 0.000777 | DOWN |
| IL22RA2 | -0.66581 | 3.145907 | 14.13653 | 0.00017 | 0.000838 | DOWN |
| NCR3 | -0.52429 | 3.337574 | 13.86346 | 0.000197 | 0.000948 | DOWN |
| IL13RA2 | 0.748131 | 3.544921 | 13.76379 | 0.000207 | 0.000992 | UP |
| CALCB | 0.943589 | 3.027226 | 13.71947 | 0.000212 | 0.001014 | UP |
| CEACAM8 | -0.87547 | 2.952888 | 13.542 | 0.000233 | 0.001099 | DOWN |
| NR1H4 | -0.94756 | 2.948773 | 13.50805 | 0.000238 | 0.001116 | DOWN |
| IFNE | 0.743116 | 2.921011 | 13.44637 | 0.000245 | 0.00115 | UP |
| CER1 | 0.601411 | 2.806973 | 13.33771 | 0.00026 | 0.001211 | UP |
| MSTN | -1.4232 | 3.439622 | 13.33621 | 0.00026 | 0.001212 | DOWN |
| KCNH2 | 0.632214 | 3.826109 | 13.23838 | 0.000274 | 0.00127 | UP |
| F2RL2 | 0.646261 | 3.336665 | 12.91958 | 0.000325 | 0.001476 | UP |
| SIGLEC8 | -0.5185 | 3.272614 | 12.87356 | 0.000333 | 0.001505 | DOWN |
| SEMA3A | 0.568955 | 4.070582 | 12.77633 | 0.000351 | 0.001575 | UP |
| ROBO2 | -0.64514 | 3.114202 | 12.77592 | 0.000351 | 0.001575 | DOWN |
| LILRA4 | -0.60051 | 3.116233 | 12.67578 | 0.00037 | 0.00165 | DOWN |
| NR1I2 | -0.85467 | 2.964618 | 12.66481 | 0.000373 | 0.001658 | DOWN |
| AMH | 0.651713 | 3.142926 | 12.53962 | 0.000398 | 0.001759 | UP |
| GLI1 | -0.59773 | 3.151485 | 12.40651 | 0.000428 | 0.001871 | DOWN |
| LEP | 0.783333 | 2.954077 | 12.38897 | 0.000432 | 0.001886 | UP |
| ESR1 | 0.554793 | 3.411147 | 12.17913 | 0.000483 | 0.002079 | UP |
| TNF | -0.50783 | 3.512603 | 11.96819 | 0.000541 | 0.002284 | DOWN |
| IFNK | 0.713149 | 2.82747 | 11.84664 | 0.000578 | 0.00242 | UP |
| IFNG | 0.550955 | 3.280706 | 11.84085 | 0.000579 | 0.002426 | UP |
| IL12RB2 | 0.599415 | 3.02005 | 11.8382 | 0.00058 | 0.002429 | UP |
| CXCL13 | -0.60081 | 6.600093 | 11.83696 | 0.000581 | 0.00243 | DOWN |
| UCHL1 | 0.710537 | 6.977871 | 11.75704 | 0.000606 | 0.002523 | UP |
| CARD18 | 0.644029 | 2.8296 | 11.74505 | 0.00061 | 0.002539 | UP |
| PTGER3 | 0.658236 | 3.03737 | 11.67578 | 0.000633 | 0.002619 | UP |
| TREML2 | -0.69151 | 3.033453 | 11.21348 | 0.000812 | 0.003252 | DOWN |
| HLA-G | -0.61884 | 5.186896 | 11.11908 | 0.000854 | 0.003397 | DOWN |
| GALP | 0.530129 | 2.803389 | 11.11729 | 0.000855 | 0.003399 | UP |
| TRIM58 | 0.724358 | 2.918956 | 10.91898 | 0.000952 | 0.003722 | UP |
| APOM | 0.503722 | 3.715218 | 10.87046 | 0.000977 | 0.003806 | UP |
| NGFR | -0.60598 | 3.382845 | 10.74319 | 0.001047 | 0.004032 | DOWN |
| TMPRSS6 | -0.7042 | 4.263695 | 10.64804 | 0.001102 | 0.004217 | DOWN |
| PTN | -0.62837 | 5.096375 | 10.52429 | 0.001178 | 0.004461 | DOWN |
| BMP3 | -0.59158 | 4.731637 | 10.52011 | 0.001181 | 0.004468 | DOWN |
| CXCL14 | -0.7936 | 8.307463 | 10.44209 | 0.001232 | 0.004639 | DOWN |
| TG | 0.637634 | 2.877527 | 10.39303 | 0.001265 | 0.004742 | UP |
| PCSK2 | -1.47757 | 5.719596 | 10.3812 | 0.001273 | 0.004768 | DOWN |
| PF4 | -0.67237 | 3.168324 | 10.36357 | 0.001285 | 0.0048 | DOWN |
| NR0B2 | -0.7683 | 4.498779 | 10.23652 | 0.001377 | 0.005094 | DOWN |
| EREG | 0.858317 | 4.799446 | 10.18737 | 0.001414 | 0.00521 | UP |
| KLK1 | 0.656012 | 3.258888 | 10.08113 | 0.001498 | 0.005481 | UP |
| HTR3C | -0.62532 | 2.839646 | 9.964847 | 0.001596 | 0.00579 | DOWN |
| NOS1 | -0.80757 | 2.965497 | 9.919235 | 0.001636 | 0.005919 | DOWN |
| TSHR | 0.632818 | 2.935679 | 9.800773 | 0.001744 | 0.006261 | UP |
| AGTR1 | 0.557202 | 3.22977 | 9.800539 | 0.001745 | 0.006261 | UP |
| EDN3 | -0.63508 | 2.848915 | 9.673929 | 0.001869 | 0.006636 | DOWN |
| TNFRSF13B | -0.53736 | 3.093367 | 9.611123 | 0.001934 | 0.006828 | DOWN |
| S100A9 | -0.64686 | 11.18639 | 9.515445 | 0.002037 | 0.007148 | DOWN |
| CSF3 | -0.69693 | 3.508432 | 9.472863 | 0.002085 | 0.007282 | DOWN |
| INSL6 | 0.851331 | 2.955953 | 9.457945 | 0.002102 | 0.007335 | UP |
| NRG4 | 0.54032 | 3.001265 | 9.437535 | 0.002126 | 0.007407 | UP |
| GLP1R | -0.67214 | 2.922711 | 8.94513 | 0.002782 | 0.009365 | DOWN |
| MC2R | 0.505535 | 2.808899 | 8.86523 | 0.002907 | 0.009712 | UP |
| ITGB3 | -0.50253 | 3.603051 | 8.832195 | 0.00296 | 0.009872 | DOWN |
| FGF18 | 0.504567 | 3.559971 | 8.828119 | 0.002966 | 0.00989 | UP |
| FGF4 | 0.554391 | 2.807848 | 8.640875 | 0.003287 | 0.010782 | UP |
| TRIM9 | 0.563642 | 3.064844 | 8.316081 | 0.00393 | 0.012533 | UP |
| PROK1 | 0.534248 | 2.821577 | 8.222656 | 0.004137 | 0.013096 | UP |
| CAMP | 0.512557 | 3.260135 | 8.187908 | 0.004217 | 0.013301 | UP |
| NLRP5 | -0.72132 | 2.853254 | 8.134788 | 0.004342 | 0.013651 | DOWN |
| CCK | -0.66758 | 2.926474 | 8.063886 | 0.004516 | 0.014111 | DOWN |
| PRL | 0.620624 | 2.847942 | 8.024661 | 0.004614 | 0.014351 | UP |
| IL19 | 0.557908 | 2.839027 | 7.755459 | 0.005355 | 0.016282 | UP |
| NDP | -0.72146 | 3.301072 | 7.540966 | 0.006031 | 0.018054 | DOWN |
| GDNF | 0.568586 | 2.952343 | 7.436471 | 0.006392 | 0.018995 | UP |
| MAPT | 0.500804 | 2.999854 | 7.391072 | 0.006555 | 0.019409 | UP |
| GLP2R | -0.70366 | 2.933331 | 7.315049 | 0.006838 | 0.020086 | DOWN |
| NR5A1 | 0.529128 | 2.836613 | 7.24078 | 0.007127 | 0.020823 | UP |
| HTR3B | 0.756615 | 2.887325 | 7.214168 | 0.007233 | 0.021065 | UP |
| PGLYRP3 | -0.6293 | 3.224974 | 7.058818 | 0.007888 | 0.022653 | DOWN |
| DUOX2 | -0.70875 | 3.963828 | 7.030734 | 0.008012 | 0.022933 | DOWN |
| FGF12 | 0.513899 | 3.146086 | 6.894925 | 0.008644 | 0.024434 | UP |
| TRIM71 | 0.559788 | 2.920482 | 6.843227 | 0.008898 | 0.025008 | UP |
| AVPR1A | 0.507905 | 3.253228 | 6.702906 | 0.009626 | 0.026785 | UP |
| NPPC | -0.578 | 2.934102 | 6.553286 | 0.010469 | 0.028711 | DOWN |
| FGF20 | 0.507454 | 2.922459 | 6.109881 | 0.013443 | 0.035632 | UP |
| FGF10 | -0.51491 | 2.886122 | 5.931826 | 0.01487 | 0.038779 | DOWN |
| GAL | -0.66947 | 4.144156 | 5.362513 | 0.020574 | 0.050899 | DOWN |
| SAA1 | -0.5013 | 6.593913 | 5.040485 | 0.024762 | 0.059466 | DOWN |
| APOH | 0.545652 | 5.213536 | 4.894372 | 0.026944 | 0.063783 | UP |
| INHA | -0.66018 | 4.982577 | 4.532834 | 0.033251 | 0.076228 | DOWN |
| KNG1 | 0.663447 | 2.998598 | 4.417134 | 0.03558 | 0.080432 | UP |
| NTS | 0.880563 | 7.73966 | 4.329475 | 0.037458 | 0.083803 | UP |
| AZGP1 | -0.50215 | 7.195578 | 3.912799 | 0.04792 | 0.102803 | DOWN |

**Supplementary Table 1.** Genes that belonged to DEGs in cluster 1 & 2 and were listed in ImmPort and InnateDB with the threshold set on P-value <0.05 and | logFC | > 0.5.
